# Evolution of DIMBOA-Glc *O*-Methyltransferases from Flavonoid *O*-Methyltransferases in the Grasses

**DOI:** 10.3390/molecules27031007

**Published:** 2022-02-02

**Authors:** Christiane Förster, Jonathan Gershenzon, Tobias G. Köllner

**Affiliations:** Department of Biochemistry, Max Planck Institute for Chemical Ecology, 07745 Jena, Germany; cfoerster@ice.mpg.de (C.F.); gershenzon@ice.mpg.de (J.G.)

**Keywords:** *O*-methyltransferase, benzoxazinoid, 5-*O*-methylflavonoid, isokaempferide, DIMBOA-Glc, FOMT2, BX10, *Zea mays*, Poaceae

## Abstract

*O*-Methylated benzoxazinoids (BXs) and flavonoids are widespread defenses against herbivores and pathogens in the grasses (Poaceae). Recently, two flavonoid *O*-methyltransferases (FOMTs), ZmFOMT2 and ZmFOMT3, have been reported to produce phytoalexins in maize (*Zea mays*). ZmFOMT2 and ZmFOMT3 are closely related to the BX *O*-methyltransferases (OMTs) ZmBX10-12 and ZmBX14, suggesting a common evolutionary origin in the Poaceae. Here, we studied the evolution and enzymatic requirements of flavonoid and BX *O*-methylation activities in more detail. Using BLAST searches and phylogenetic analyses, we identified enzymes homologous to ZmFOMT2 and ZmFOMT3, ZmBX10-12, and ZmBX14 in several grasses, with the most closely related candidates found almost exclusively in species of the Panicoideae subfamily. Biochemical characterization of candidate enzymes from sorghum (*Sorghum bicolor*), sugar cane (*Saccharum spp.*), and teosinte (*Zea nicaraguensis*) revealed either flavonoid 5-*O*-methylation activity or DIMBOA-Glc 4-*O*-methylation activity. However, DIMBOA-Glc 4-OMTs from maize and teosinte also accepted flavonols as substrates and converted them to 3-*O*-methylated derivatives, suggesting an evolutionary relationship between these two activities. Homology modeling, sequence comparisons, and site-directed mutagenesis led to the identification of active site residues crucial for FOMT and BX OMT activity. However, the full conversion of ZmFOMT2 activity into BX OMT activity by switching these residues was not successful. Only trace *O*-methylation of BXs was observed, indicating that amino acids outside the active site cavity are also involved in determining the different substrate specificities. Altogether, the results of our study suggest that BX OMTs have evolved from the ubiquitous FOMTs in the PACMAD clade of the grasses through a complex series of amino acid changes.

## 1. Introduction

*O*-Methylation of plant specialized metabolites is a widespread biochemical transformation that creates structural diversity and forms products with modified chemical properties that help plants cope with different biotic and abiotic stresses. This tailoring reaction is catalyzed by *S*-adenosyl-l-methionine (SAM)-dependent *O*-methyltransferases (OMTs), which transfer the methyl group of the cosubstrate SAM to a hydroxyl moiety of various acceptor molecules. The methyl ether derivatives produced often exhibit reduced reactivity of hydroxyl groups and altered solubility, which in turn affects their biological activity, stability, intracellular localization, and metabolic fate [1,2]. *O*-Methylated specialized metabolites can fulfill important roles in plant defense by acting, for example, as lignin precursors or as induced antimicrobial compounds [3,4].

Plant OMTs have been classified into three major groups based on phylogenetic analysis, conserved amino acid sequence features, protein structures, and substrate specificities: caffeic acid OMTs (COMTs), caffeoyl-CoA OMTs (CCoAOMTs), and carboxylic acid OMTs [5,6,7]. The latter share only marginal sequence similarities with the other classes of OMTs and are involved, for instance, in the methylation of various organic acids, such as salicylic acid, jasmonic acid, or indole-3-acetic acid [8]. CCoAOMTs are bivalent cation-dependent enzymes that mainly methylate coenzyme A (CoA) esters of phenylpropanoids to lignin precursors. All remaining plant OMTs belong to the large COMT class, which accepts a variety of different substrates, including phenylpropanoids, flavonoids, alkaloids, and coumarins. COMTs are larger (40–43 kDa) than CCoAOMTs (26–30 kDa) and do not require bivalent cations for activity [6,9]. The majority of plant OMTs show remarkable substrate specificity as well as regiospecificity for certain hydroxyl groups [2]. However, multifunctional enzymes that are able to utilize structurally related compounds [10,11] or exhibit nonselective positional activity [12,13,14] have also been described. In some cases, substrate specificity or regiospecificity has been associated with a single amino acid change [15,16].

In the grasses (Poaceae), OMTs are involved, among other processes, in the biosynthesis of two important classes of defense compounds: flavonoids and benzoxazinoids (BXs). Flavonoids and their *O*-methyl derivatives are ubiquitously distributed in the plant kingdom and constitute one of the largest groups of plant natural products [17,18]. The flavonoid core structure is derived from the acetate and phenylpropanoid pathways and can subsequently undergo numerous modifications [19], including *O*-methylation by flavonoid OMTs (FOMTs). *O*-Methylated flavonoids have been described as potent antimicrobial phytoalexins. For example, sakuranetin (7-*O*-methylnaringenin), produced by the FOMT OsNOMT in rice (*Oryza sativa*) leaves upon fungal infection or abiotic stress, inhibits the growth of rice blast fungus (*Pyricularia oryzae*) in vivo and shows antifungal activity in vitro [20,21,22,23,24]. Similarly, pathogen-induced formation of *O*-methylflavonoids was also detected in other grasses, such as sorghum (*Sorghum bicolor*), maize (*Zea mays*), and barley (*Hordeum vulgare*) [25,26,27]. Recently, we characterized four FOMTs from maize, ZmFOMT2 and ZmFOMT3, ZmFOMT4, and ZmFOMT5, that methylate the hydroxyl groups of various flavonoids regiospecifically at positions 5, 7, and 6 of the A ring, respectively, and are involved in the fungus-induced formation of complex *O*-methylflavonoid mixtures [28].

Unlike flavonoids, BXs are mostly restricted to the grass family and a few dicotyledonous species [29,30]. The BX biosynthetic pathway branches off from tryptophan biosynthesis and has been elucidated in maize [29,31,32]. *O*-Methylated BXs usually show higher activity than their non- or less *O*-methylated precursors. 2-Hydroxy-4,7-dimethoxy-1,4-benzoxazin-3-one glucoside (HDMBOA-Glc), for example, was demonstrated to be more effective in deterring and reducing the growth of chewing herbivores [33,34] and to also be more toxic to phloem-feeding aphids compared to its precursor 2,4-dihydroxy-7-methoxy-1,4-benzoxazin-3-one glucoside (DIMBOA-Glc) [31,35]. The conversion of DIMBOA-Glc to HDMBOA-Glc is induced upon herbivory, fungal infection, or elicitor treatment in maize and other grasses [33,36,37,38], and four OMTs, ZmBX10, ZmBX11, ZmBX12, and ZmBX14, have been described to catalyze this reaction in maize [31,32]. Notably, DIMBOA-Glc 4-OMT activity was also found in wheat; however, the corresponding enzyme TaBX10 is not related to ZmBX10-12 and ZmBX14, indicating the independent evolution of DIMBOA-Glc 4-OMT activity in maize and wheat [39]. Despite extensive knowledge on the biosynthesis and biological activity of *O*-methylated BXs, the evolutionary origin of the OMTs that produce them remains unclear [40].

In this study, we investigated the evolution of DIMBOA-Glc 4-OMTs in maize. Because the recently identified flavonoid 5-OMTs ZmFOMT2 and ZmFOMT3 are closely related to ZmBX10-12 and ZmBX14 [28], we hypothesized that DIMBOA-Glc 4-OMT activity evolved from FOMT activity in the PACMAD clade of the grasses, one of the two major clades of Poaceae, named after the subfamilies Panicoideae, Aristidoideae, Chloridoideae, Micrairoideae, Arundinoideae, and Danthonioideae [41]. To investigate this evolutionary scenario, we performed phylogenetic analyses to identify ZmFOMT2 homologs in several Poaceae species, followed by in vitro enzyme characterization. In addition, comparative sequence analyses, homology modeling, and in vitro mutagenesis allowed us to examine amino acid residues involved in the catalysis of flavonoid 5-OMTs and DIMBOA-Glc 4-OMTs. Our results suggest that DIMBOA-Glc OMTs evolved from FOMTs in the PACMAD clade of the grasses and that this change in activity was due to a complex series of amino acid mutations.

## 2. Results

### 2.1. Close Homologs of the Flavonoid 5-OMT ZmFOMT2 Are Restricted to the Panicoideae Subfamily

**Figure 1:**
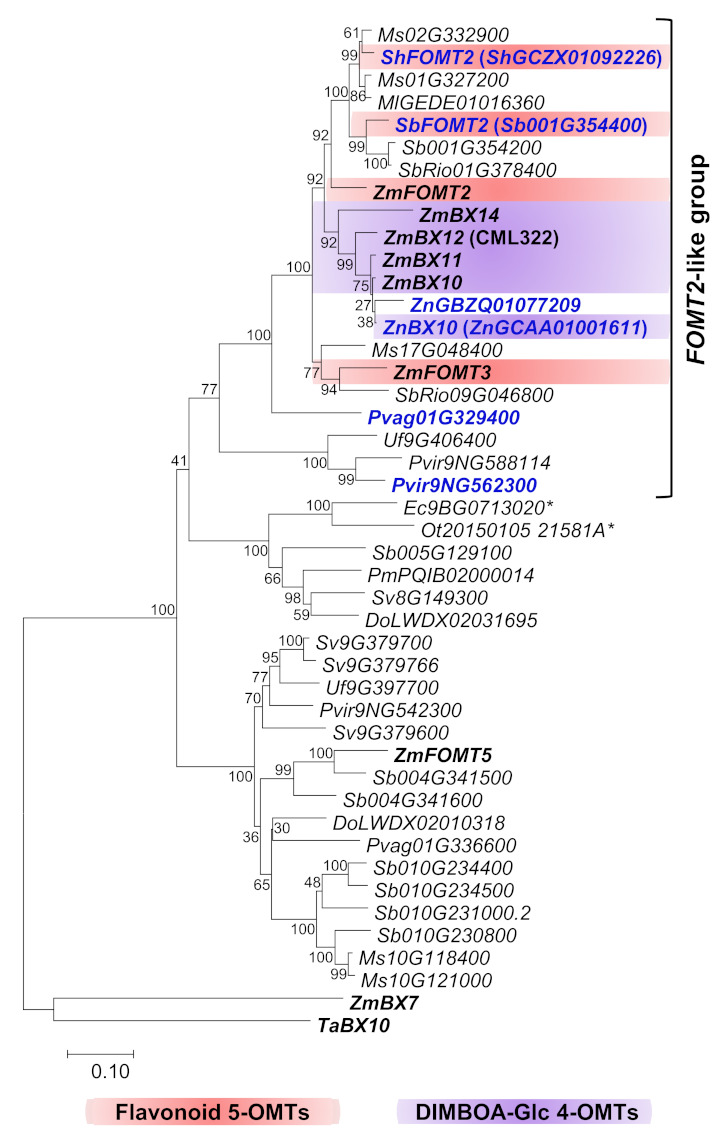
Phylogenetic analysis of putative FOMT and BX OMT genes similar to *ZmFOMT2* in diverse species of the Poaceae. The tree represents a subtree (“PACMAD-specific *FOMT2-BX10* clade”) of a larger tree given in Figure S1. The tree was inferred using the maximum likelihood method based on the general time-reversible model, including gamma-distributed rate variation among sites (+G, 1.2811). Bootstrap values (*n* = 1000) are shown next to each node. The tree is drawn to scale, with branch lengths measured in the number of substitutions per site. All positions with < 80% site coverage were eliminated. *ZmBX7* (*Zm00001d049179*) and *TaBX10* (*4AL_C467B516F*) were used as an outgroup for rooting. OMTs investigated in this study are highlighted in bold blue text, and previously characterized genes are shown in bold black text. Genes marked with (*) belong to the Chloridoideae subfamily, and all other genes belong to the Panicoideae subfamily. Species abbreviations: Do, *Dichanthelium oligosanthes*; Ec, *Eleusine coracana*; Ml, *Miscanthus lutarioriparius*; Ms, *Miscanthus sinensis*; Ot, *Oropetium thomaeum*; Pm, *Panicum miliaceum*; Pvir, *Panicum virgatum*; Pvag, *Paspalum vaginatum*; Sh, *Saccharum* *hybrid*; Sb, *Sorghum bicolor*; SbRio, *Sorghum bicolor* Rio; Sv, *Setaria viridis*; Ta, *Triticum aestivum*; Uf, *Urochloa fusca*; Zm, *Zea mays*; Zn, *Zea nicaraguensis*.

To identify *ZmFOMT2* homologs in the Poaceae family, we performed comprehensive BLAST and phylogenetic analyses including almost all Poaceae genomes available in the Phytozome 13 and NCBI databases (Figure 1 and Supplementary Materials Figure S1). The resulting phylogenetic tree showed that the closely related maize DIMBOA-Glc 4-OMT genes *ZmBX10-12* and *ZmBX14*, the flavonoid 5-OMT genes *ZmFOMT2* and *ZmFOMT3*, and the flavonoid 6-OMT gene *ZmFOMT5* clustered together with putative COMT genes from several other species in a well-defined subclade that we designated the “PACMAD-specific *FOMT2-BX10* clade”. This subclade is depicted in Figure 1, and the entire tree is shown in Figure S1. The *FOMT2-BX10* subclade consists entirely of genes from species belonging to Panicoideae and Chloridoideae within the major PACMAD grass clade, with a clear preponderance of Panicoideae genes (Figure 1). To select the most relevant candidate genes, we subdivided this subtree and focused only on its upper part (hereafter referred to as the “*FOMT2*-like group”), which contains COMTs with 60–87% amino acid sequence identity to ZmFOMT2. The previously characterized wheat DIMBOA-Glc 4-OMT gene *TaBX10* [39], on the other hand, clustered in a clearly separated subclade, the “BOP-specific *BX10* clade”; Figure S1) named for the subfamilies Bambusoideae, Oryzoideae, and Pooideae [41].

### 2.2. ZmFOMT2 Homologs Have DIMBOA-Glc 4-OMT or Flavonoid 5-OMT Activity

Six putative COMT genes that clustered in different subclades of the *FOMT2*-like group (Figure 1) were chosen for biochemical characterization. The complete open reading frames (ORFs) of *ShGCZX01092226* (from a *Saccharum* hybrid), *Sb001G354400* (from *Sorghum bicolor*), *ZnGBZQ01077209* (*ZnGBZQ*, from *Zea nicaraguensis,* teosinte), *ZnGCAA01001611*, *Pvag01G329400* (*Pvag9400*, from *Paspalum vaginatum*), and *Pvir9NG562300* (from *Panicum virgatum*) were either amplified from cDNA and cloned or synthesized. Because the cloned ORF of *Pvir9NG562300* had only 98% amino sequence identity with its respective database sequence, it was designated *Pvir2300-like.* ZnGCAA01001611, ShGCZX01092226, and Sb001G354400 were designated ZnBX10, ShFOMT2, and SbFOMT2, respectively, according to their in vitro activity described below.

**Figure 2:**
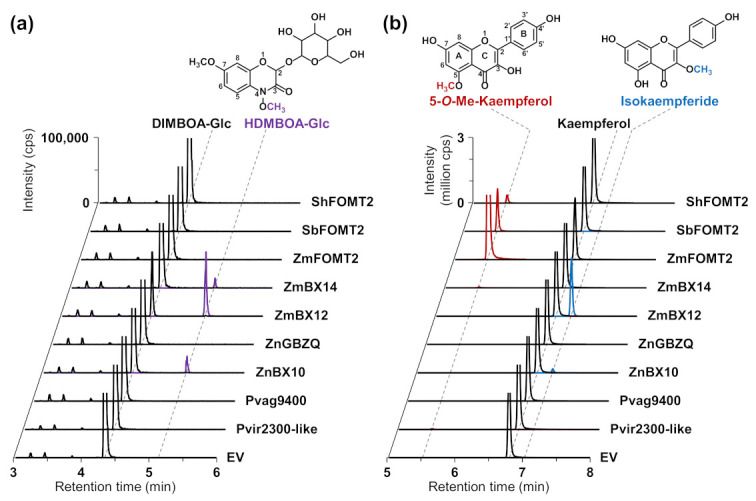
Activity of enzymes in the *FOMT2*-like group. The purified recombinant enzymes as well as an empty vector control (EV) were incubated with the potential substrates DIMBOA-Glc (**a**) and kaempferol (**b**) in the presence of the cosubstrate *S*-adenosyl-l-methionine (SAM). Reaction products were analyzed by LC–MS/MS. Chromatograms of specific MRM transitions (see Methods section) are shown. In the upper part, the structures of the enzymatic products are shown with the attached methyl groups highlighted in purple (HDMBOA-Glc), red (5-*O*-methylkaempferol), and blue (isokaempferide), respectively. All assays were performed in technical triplicates. Abbreviations: Me—methyl; cps—counts per second.

To test for enzymatic activity, all genes were heterologously expressed in *Escherichia coli*, and the purified His-tagged proteins were incubated with DIMBOA-Glc or different flavonoids as potential substrates in the presence of the cosubstrate SAM. Product formation was analyzed using liquid chromatography–tandem mass spectrometry (LC–MS/MS). The previously characterized flavonoid 5-OMT ZmFOMT2 and DIMBOA-Glc 4-OMTs ZmBX12 and ZmBX14 served as positive controls. Besides ZmBX14 and ZmBX12, ZnBX10 also converted DIMBOA-Glc to HDMBOA-Glc (Figure 2a, Table S1), while no product peak was observed in the empty vector (EV) control. However, DIMBOA-Glc was not accepted as a substrate by ZnGBZQ, the second enzyme from teosinte studied, or any other of the putative COMTs in the *FOMT2*-like group (Figure 2a, Table S1).

### 2.3. The DIMBOA-Glc 4-OMTs ZmBX10-12 and ZnBX10 also Possess Flavonoid 3-OMT Activity with Flavonols

In a previous study, we showed that ZmBX10-12 and ZmBX14 exhibited nonspecific trace activity with various flavonoids, such as naringenin, apigenin, and scutellarein [28]. However, when the flavonol kaempferol, which, in contrast to the above-mentioned flavonoids, contains an additional hydroxyl group at position 3 of the C ring, was tested as a substrate, ZmBX10-12 and ZnBX10 produced substantial amounts of the 3-*O*-methylated derivative isokaempferide (Figure 2b and Figure S2; Table S1), while ZmFOMT2 and SbFOMT2 produced only trace amounts of this compound (Table S1). Notably, the catalytic efficiency of the BX OMTs towards kaempferol followed the same order as shown for DIMBOA-Glc [28,31], with ZmBX12 as the most active OMT, followed by ZmBX10, ZmBX11, and ZmBX14 (Figure S2). The flavonoid 6-OMT ZmFOMT5 [28] also accepted kaempferol as a substrate and produced minor amounts of isokaempferide (Figure S2). Untargeted LC–MS measurements with accurate mass determination showed that ZmBX12 and ZnBX10 were also active with quercetin, a flavonol similar to kaempferol, and both the MS/MS fragmentation pattern and the LC elution order indicated methylation at the 3-hydroxyl group of the C ring (Figure S3).

### 2.4. Identification of Active Site Residues Determining the Substrate Specificities and Activities of Flavonoid 5-OMTs and DIMBOA-Glc 4-OMTs

**Figure 3:**
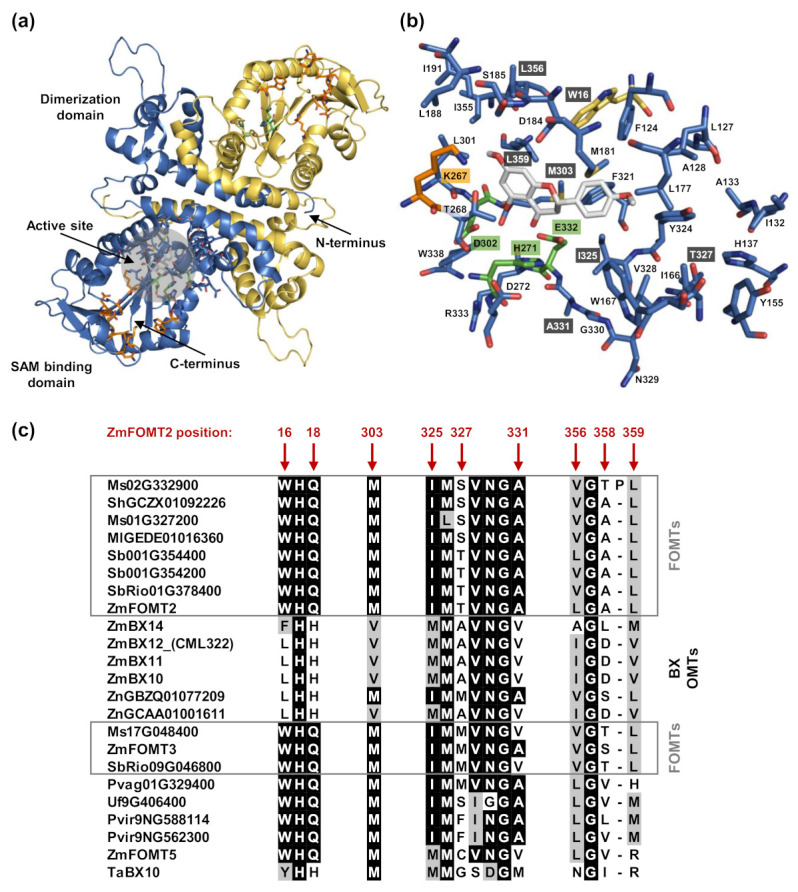
Structural model of ZmFOMT2 and alignment-based identification of putative active site residues. (**a**) Homology model of the biological homodimer of ZmFOMT2 (chain A: blue; chain B: gold) based on the template isoflavone 4′-OMT from *Medicago truncatula* (MtHI4OMT; PDB-ID: 1ZG3). Labels correspond to the monomer colored in blue. A close-up view of the putative active site is shown in (**b**), representing the 6 Å area around naringenin, docked in as a model flavonoid substrate. Amino acid residues (carbon atoms) are color-coded, with the catalytic triad in green, SAM binding residues in orange, naringenin in gray, and all other protein carbon atoms in blue (chain A) and gold (chain B). Oxygen atoms are red, nitrogen atoms are blue, and sulfur atoms are yellow. Mutation sites are labeled in dark gray. (**c**) Alignment of putative active site residues that differ between all ZmFOMT2-like FOMTs (gray boxes) and BX OMTs, respectively. The amino acid sequence sections are cut from the complete alignment given in Figure S5. Identical amino acids are shaded in black, and similar amino acids are in gray.

**Figure 4:**
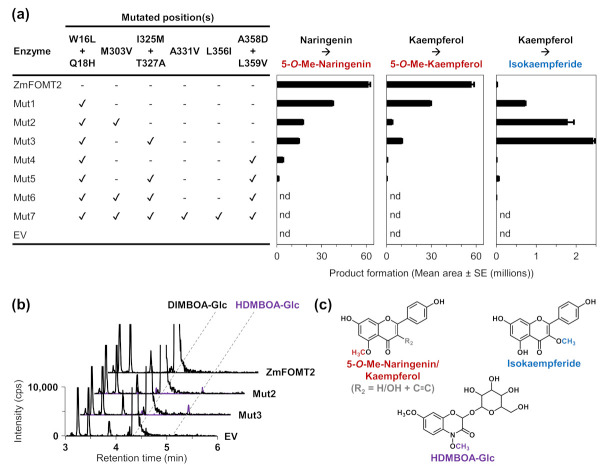
Enzymatic activity of ZmFOMT2 mutants with flavonoids and DIMBOA-Glc. The purified recombinant enzymes as well as an empty vector control (EV) were incubated with the substrates naringenin or kaempferol (**a**) and DIMBOA-Glc (**b**) in the presence of the cosubstrate SAM. Reaction products were analyzed by LC–MS/MS. Product formation is shown in bar charts (mean area ± SE (*n* = 3)) or as chromatograms of specific MRM transitions (see Methods section). In contrast to the assays shown in panel (**a**), for the assays in panel (**b**), a 2.5-fold greater amount of purified recombinant enzyme (~2 µg/assay), a 2.5-fold greater amount of substrate (50 µM), and a 5-fold greater amount of cosubstrate were used. (**c**) Structures of the enzymatic products with the attached methyl groups are highlighted in red (5-*O*-methylnaringenin and 5-*O*-methylkaempferol), blue (isokaempferide), and purple (HDMBOA-Glc). Abbreviations: Me—methyl; nd—not detected; cps—counts per second.

To identify amino acid residues that determine flavonoid 5-OMT and DIMBOA-Glc 4-OMT activity, respectively, we conducted homology modeling of ZmFOMT2 and amino acid sequence comparisons among all enzymes in the *FOMT2*-like group (Figure 3). The crystal structure of an isoflavone 4′-OMT (MtHI4OMT) from *Medicago truncatula* [42], which shares 39% amino acid sequence identity with ZmFOMT2, was used as a template for the construction of the ZmFOMT2 model (Figure 3a and Figure S4). Docking of naringenin into the substrate binding pocket of ZmFOMT2 revealed 37 amino acids that were located at a distance of ≤6 Å around the docked substrate (Figure 3b, Table S2). Nine of these residues differed between all functional flavonoid 5-OMTs and DIMBOA-Glc 4-OMTs (Figure 3c and Figure S5) and were therefore further analyzed by replacing them in ZmFOMT2 with the corresponding residues of ZmBX10. To reduce the number of mutants to be generated, nearby residues were combined in a single mutation step, resulting in three different double mutations and three single mutations (Figure 3c). When fed with naringenin or kaempferol as substrate, the double mutant ZmFOMT2 W16L + Q18H, the triple mutant ZmFOMT2 W16L + Q18H + M303V, and the quadruple mutant ZmFOMT2 W16L + Q18H + I325M + T327A still showed flavonoid 5-OMT activity; however, there was a successive decrease in activity compared to the wild-type ZmFOMT2 enzyme (Figure 4a). At the same time, successively increased production of isokaempferide from kaempferol was observed (Figure 4a). The introduction of two further mutations (A358D + L359V) into the quadruple mutant ZmFOMT2 W16L + Q18H + I325M + T327A almost completely abolished any enzymatic activity, and the simultaneous mutation of all nine residues also led to an inactive protein (Figure 4a). Notably, none of the tested mutants was able to accept DIMBOA-Glc as substrate in our standard assay. However, when using 2.5-fold greater amounts of both the purified recombinant enzymes and DIMBOA-Glc and a 5-fold greater amount of SAM, the triple mutant ZmFOMT2 W16L + Q18H + M303V and the quadruple mutant ZmFOMT2 W16L + Q18H + I325M + T327A produced small amounts of HDMBOA-Glc, while neither the EV control nor ZmFOMT2 showed any DIMBOA-Glc 4-OMT activity (Figure 4b).

## 3. Discussion

*O*-Methylated flavonoids and BXs are important anti-pathogen and anti-herbivore defense compounds widespread in the grasses [22,33,37,39]. Recently, we identified two flavonoid 5-OMTs in maize, ZmFOMT2 and ZmFOMT3, and showed that they are closely related to the DIMBOA-Glc 4-OMTs ZmBX10-12 and ZmBX14, indicating a common OMT ancestor in the grasses [28]. To investigate the evolution of these enzymes in more detail, we characterized OMTs homologous to ZmFOMT2 from several other grass species. Our studies support the hypothesis that maize and teosinte DIMBOA-Glc 4-OMTs originated from FOMTs in the PACMAD clade. In addition, we identified amino acid residues in the active site of ZmFOMT2 that, when mutated to the corresponding residues of ZmBX10-12, altered the regiospecificity and catalytic activity of the enzyme.

The recently discovered DIMBOA-Glc 4-OMT gene *TaBX10* in wheat was shown not to be orthologous to *ZmBX10-12 and ZmBX14* in maize. Therefore, DIMBOA-Glc 4-OMT activity is thought to have evolved independently in maize and wheat [39]. Our phylogenetic analyses, encompassing a larger set of Poaceae species, are consistent with this finding, as close homologs of *ZmFOMT2, ZmBX10-12, and ZmBX14* were found exclusively in the PACMAD clade, with the majority of genes belonging to the Panicoideae subfamily (PACMAD-specific *FOMT2-BX10* clade; Figure 1 and Figure S1). Biochemical characterization of selected candidate enzymes revealed good agreement between grouping within this subclade and actual OMT activity (Figure 2 and Figure S2; Table S1). While ShFOMT2 and SbFOMT2 clustered with ZmFOMT2 and exhibited flavonoid 5-OMT activity, ZnBX10 clustered with ZmBX10-12 and ZmBX14 and showed DIMBOA-Glc 4-OMT activity. In contrast, Pvag01G329400 and Pvir2300-like clustered in smaller and more basal subclades of the phylogenetic tree and showed negligible or no flavonoid 5-OMT activity (Figure 1, Figure 2 and Figure S2; Table S1). However, whether these two enzymes accept other phenylpropanoids as substrates remains to be determined. 

Notably, DIMBOA-Glc 4-OMTs could only be identified in maize and its wild relative teosinte and not in the other species investigated, which is consistent with the distribution of BXs in these grasses [30]. Overall, our findings suggest that flavonoid 5-OMT activity is more widespread than DIMBOA-Glc 4-OMT activity, at least in the Panicoideae, and that DIMBOA-Glc 4-OMTs evolved from a flavonoid 5-OMT ancestor. Whether the wheat DIMBOA-Glc 4-OMT TaBX10 also evolved from an FOMT ancestor enzyme is still unclear. Thus, further studies are necessary to understand how these important plant defense genes have evolved independently in the grasses.

COMTs are known to generally catalyze the *O*-methylation of a variety of structurally diverse substrates; however, closely related COMTs often use structurally similar substrates [6]. BXs and flavonoids indeed share a similar basic chemical skeleton consisting of three six-membered rings, with the central ring being a heterocycle (Figure 2). Our results showed that the DIMBOA-Glc 4-OMTs, ZmBX10-12 and ZnBX10, are able to *O*-methylate the flavonols kaempferol and quercetin at position 3 of the C ring, which is close to position 4 in BXs (Figure 2, Figures S2 and S3; Table S1). This indicates a similar orientation and binding mode for DIMBOA-Glc and flavonoid substrates in the active sites of these enzymes. Previous publications showed that the substrate preference, regiospecificity, and overall activity of FOMTs can be altered by a few amino acid mutations in the substrate binding pocket [16,43,44]. Using homology modeling and amino acid sequence comparisons, we identified nine putative active site residues that differ between flavonoid 5- and DIMBOA-Glc 4-OMTs (Figure 3, Figures S4 and S5). Unexpectedly, the replacement of some of these residues in ZmFOMT2 with the corresponding residues of ZmBX10 only produced very weak BX OMT activity, whereas the complete replacement of these residues resulted in an inactive enzyme. It thus appears that additional mutations are required for the switch from flavonoid 5-OMT to DIMBOA-Glc 4-OMT activity (Table S3). Indeed, studies on terpene synthases from tobacco and maize have shown that amino acid residues near but not in the active site can also play a role in determining catalytic specificities [45,46]. 

Interestingly, the stepwise and additive mutation of the nine identified active site residues in ZmFOMT2 led to mutants with altered catalytic efficiencies and substrate regiospecificities for flavonoids. While wild-type ZmFOMT2 was highly regiospecific for position 5 on the A ring of flavonoids, even when using flavonols such as kaempferol or quercetin as substrates, the double mutant ZmFOMT2 W16L + Q18H showed flavonol 3-OMT activity at the expense of flavonoid 5-OMT activity (Figure 4). This effect was even stronger in the triple mutant ZmFOMT2 W16L + Q18H + M303V and the quadruple mutant ZmFOMT2 W16L + Q18H + I325M + T327A. Moreover, the triple mutant ZmFOMT2 W16L + Q18H + M303V and the quadruple mutant ZmFOMT2 W16L + Q18H + I325M + T327A both produced trace amounts of HDMBOA-Glc (Figure 4), indicating that the identified active site residues are involved in substrate binding and positioning. Previous studies [28,31] and the biochemical data presented here show that the DIMBOA-Glc 4-OMTs and the flavonoid 5-OMTs exhibit different catalytic efficiencies despite generally comparable enzymatic functionality (Figure 2, Figures S2 and S3; Table S1). Altogether, the results of our study suggest that the evolution of DIMBOA-Glc 4-OMTs involved complex mutations that not only altered substrate specificity but also fine-tuned catalytic efficiency. Follow-up studies based on structural data from a plant OMT more closely related to ZmFOMT2 are needed to unravel the enzymatic requirements for the substrate specificity of flavonoid 5-OMT and DIMBOA-Glc 4-OMT in more detail.

## 4. Materials and Methods

### 4.1. Plants and Growth Conditions

Seeds of *Sorghum bicolor* (L.) Moench subsp. bicolor race bicolor ‘Lisorax’ and *Panicum virgatum* L. were provided by the Leibniz-Institut für Pflanzengenetik und Kulturpflanzenforschung (IPK, Gatersleben, Germany). Plants were potted in soil (mix of 70 L Tonsubstrat with 200 L Kultursubstrat TS 1, Klasmann-Deilmann, Geeste, Germany) and grown in the greenhouse for two weeks.

### 4.2. RNA and cDNA Preparation

Total RNA was extracted from approximately 50 mg frozen plant powder using the InviTrap Spin Plant RNA Kit (STRATEC, Birkenfeld, Germany) according to the manufacturer’s instructions. The RNA concentration and purity were assessed with a spectrophotometer (NanoDrop 2000c, Thermo Fisher Scientific, Schwerte, Germany). RNA (1 µg) was treated with DNaseI (Thermo Fisher Scientific), followed by cDNA synthesis using SuperScript III reverse transcriptase and oligo (dT)_20_ primers (Invitrogen by Thermo Fisher Scientific) according to the manufacturer’s instructions.

### 4.3. Gene Synthesis

The complete ORFs of *ShGCZX01092226* (*ShFOMT2*), *ZnGBZQ01077209*, *ZnGCAA01001611* (*ZnBX10*), and *Pvag01G329400* were synthesized after codon optimization for heterologous expression in *E. coli* and subcloned into the expression vector pET100/D-TOPO using the GeneArt gene synthesis and express cloning service (Thermo Fisher Scientific) (for sequences, see Figure S6).

### 4.4. Site-Directed Mutagenesis

For in vitro mutagenesis, 10–50 ng pET100/D-TOPO vector harboring the *ZmFOMT2* ORF was used as template for 18 cycles of mutagenesis PCR using Q5 High-Fidelity DNA Polymerase (New England Biolabs, Frankfurt (Main), Germany) and the primers listed in Table S4. The primers used contained the desired mutations, and the pairs were designed with either completely overlapping or only partially overlapping sequences, the latter allowing enhanced amplification efficiency from a smaller amount of plasmid template [47]. After PCR amplification, the plasmid template was digested with *DpnI*. The mutagenized PCR product was purified using the QIAquick PCR Purification Kit (QIAGEN, Hilden, Germany) according to the manufacturer’s instructions and transferred into *E. coli* 10-beta cells (New England Biolabs) for recovery and amplification. All mutagenized plasmids were fully sequenced.

### 4.5. Cloning and Heterologous Expression of OMT Genes in E. coli

The full-length ORFs of *Sb001G354400* (*SbFOMT2*) and *Pvir9NG562300* (*Pvir2300-like*) were amplified from cDNA derived from young leaves of *S. bicolor* and *P. virgatum*, respectively, with the primer pairs listed in Table S4. The resulting PCR products were cloned into the expression vector pET100/D-TOPO (Invitrogen by Thermo Fisher Scientific) or pASK-IBA37plus (IBA Lifesciences, Göttingen, Germany) and fully sequenced. *ZmFOMT2* (W22; GenBank accession: MZ484743) was provided as a pET100/D-TOPO construct, while *ZmBX10* (B73), *ZmBX11* (B73), *ZmBX12* (CML322), *ZmBX14* (B73), and *E. coli* codon-optimized *ZmFOMT5* were available as pASK-IBA37plus constructs from our previous studies [28,31,32]. All OMTs were heterologously expressed in the *E. coli* strain BL21 (DE3) (Invitrogen by Thermo Fisher Scientific) as previously described [28]. Liquid cultures were grown in lysogeny broth at 37 °C and 220 rpm, induced at an OD_600_ of 0.8 with a final concentration of 1 mM IPTG (pET100/D-TOPO) or 200 µg/L anhydrotetracycline (pASK-IBA37plus), and subsequently incubated at 18 °C and 220 rpm for 15 h. The cells were harvested by centrifugation (5000 g, 4 °C, 10 min), resuspended in refrigerated extraction buffer (50 mM Tris-HCl pH 8, 500 mM NaCl, 20 mM imidazole, 10% (*v/v*) glycerol, 1% (*v/v*) Tween 20, and 25 U/mL freshly added Benzonase Nuclease (Merck, Darmstadt, Germany)), and disrupted by sonication (4 × 20 s; Bandelin UW 2070, Berlin, Germany). Afterwards, cell debris was removed by centrifugation (16,000 g, 4 °C, 20 min), and the N-terminal His-tagged proteins were purified from the supernatant using HisPur Cobalt Spin Columns (Thermo Fisher Scientific) according to the manufacturer’s instructions. Tris-HCl buffer (pH 8, without Tween 20; see above) containing either 20 mM or 250 mM imidazole was used for equilibration/washing and elution steps, respectively. The purified proteins were desalted by gel filtration using illustra NAP Columns (GE Healthcare, Freiburg, Germany) and eluted in assay buffer (50 mM Tris-HCl pH 7, 10% (*v/v*) glycerol). Alternatively, Amicon Ultra-0.5 centrifugal filter devices (Merck) were used for concentration and desalting of the His-purified proteins. Protein concentrations were determined by the Bradford method using Quick Start™ Bradford 1× Dye Reagent (Bio-Rad Laboratories, Feldkirchen, Germany) and prediluted BSA protein standards (Thermo Fisher Scientific) in a 96-well microtiter plate. The measurements were performed on a Tecan infinite 200 microplate reader (TECAN, Männedorf, Switzerland) using Magellan software (TECAN) for instrument control and data analysis.

### 4.6. In Vitro Enzyme Assays

To test OMT activities, assays were conducted as previously described [28]. Briefly, the 100 µL assay mixtures contained 500 µM dithiothreitol (DTT), 100 µM of the cosubstrate *S*-adenosyl-L-methionine (SAM), 20 µM of the substrate (DIMBOA-Glc or various flavonoids), and 0.8 µg purified recombinant protein in assay buffer (50 mM Tris-HCl, pH 7, 10% (*v/v*) glycerol). All assays were incubated for 1 h at 25 °C and stopped by adding one volume of 100% methanol. Denatured proteins were removed by centrifugation (4000 g, 5 min), and product formation was monitored by the analytical methods described below. 

### 4.7. Liquid Chromatography–Mass Spectrometry (LC–MS) Analysis of BXs and Flavonoids in Enzyme Assays

#### 4.7.1. Targeted LC–MS/MS Analysis

The analysis of BXs and flavonoids was performed as described previously in Förster et al. (2021). Briefly, an Agilent 1260 Infinity II LC system (Agilent Technologies, Frankfurt (Main), Germany) coupled to a QTRAP 6500+ tandem mass spectrometer (Sciex, Darmstadt, Germany) was used for the analysis. Chromatographic separation was achieved on a ZORBAX Eclipse XDB-C18 column (50 × 4.6 mm, 1.8 μm; Agilent Technologies) using a 1.1 mL/min flow rate. Aqueous formic acid (0.05% (*v/v*)) and acetonitrile were used as mobile phases A and B, respectively. The following gradients were used: BXs: 0 to 0.5 min, 5% B; 0.5 to 6.0 min, 5 to 32.5% B; 6.02 to 7.0 min, 100% B; 7.10 to 9.5 min, 5% B; flavonoids: 0 to 0.5 min, 10% B; 0.5 to 8.0 min, 10 to 55% B; 8.5 to 9.0 min, 100% B; 9.02 to 11 min, 10% B. The column temperature was maintained at 20°C. The injection volume was 4 µL for enzyme assays with DIMBOA-Glc as substrate and 1 µL for enzyme assays with flavonoid substrates. The mass spectrometer was equipped with a turbospray ESI ion source, operated in negative or positive ionization mode, for the analysis of BXs and flavonoids, respectively (detailed parameters are provided in Table S5). Multiple reaction monitoring (MRM) was used to monitor analyte parent ion → product ion transitions as listed in Table S6. Product identities were confirmed by authentic standards or deduced from specific enzymatic activities as described previously in Förster et al. (2021). For data acquisition and processing, Analyst 1.6.3 (Sciex) and MultiQuant 3.0.3 software (Sciex) were used.

#### 4.7.2. Untargeted LC–MS Analysis with Accurate Mass Determination

To screen for potential unknown enzymatic products, untargeted LC–MS was used as previously described [28]. Chromatography was performed on a Dionex UltiMate 3000 RS pump system (Thermo Fisher Scientific) equipped with a ZORBAX RRHD Eclipse XDB-C18 column (2.1 × 100 mm, 1.8 µm; Agilent Technologies), using aqueous formic acid (0.1% (*v/v*)) and acetonitrile as mobile phases A and B, respectively. The flow rate was 0.3 mL/min and the column temperature was maintained at 25 °C. The elution profile was as follows: 0 to 0.5 min, 5% B; 0.5 to 11 min, 5 to 60% B; 11.1 to 12 min, 100% B; 12.1 to 15 min, 5% B. The injection volume was 4 µL. The LC system was coupled to a timsTOF mass spectrometer (Bruker Daltonics, Bremen, Germany) equipped with an ESI ion source, operated in positive ionization mode to scan masses from *m/z* 50 to 1500. The MS settings were as follows: capillary voltage, 4500 V; drying gas (nitrogen), 8 L/min, 280 °C; nebulizer gas (nitrogen), 2.8 bar. In autoMS/MS mode, alternating collision energy (20/50 eV) was applied. Internal calibration was achieved using sodium formate adducts. Bruker oTOF control 6.0.115 and HyStar 5.1.8.1 software (Bruker Daltonics) were used for data acquisition, and DataAnalysis 5.3 (Bruker Daltonics) was used for data processing.

### 4.8. Sequence Alignment and Phylogenetic Analysis

OMTs were identified by BLASTP analysis with ZmFOMT2 as the query and using Poaceae protein datasets available in the Phytozome 13 (https://phytozome-next.jgi.doe.gov/ accessed on 16 April 2021; for all datasets used, see Table S7) and NCBI (https://www.ncbi.nlm.nih.gov/ accessed on 24 March 2021) databases. In addition, a local BLAST was performed with transcriptomic data from the NCBI Transcriptome Shotgun Assembly (TSA, https://www.ncbi.nlm.nih.gov/genbank/tsa/ accessed on 25 March 2021) sequence database using the program BioEdit [48], and the full sequences of the resulting hits were retrieved from the NCBI database. Only genes (ORFs) with ≥80% query coverage and a corresponding amino acid identity of ≥40% were used for phylogenetic analysis. All sequences with ≤5 amino acid differences were excluded. Multiple sequence alignments were computed using the MUSCLE codon algorithm implemented in the software MEGA7 [49]. Based on these alignments, phylogenetic trees were reconstructed with MEGA7 using a maximum likelihood algorithm. Codon positions included were 1st+2nd+3rd+noncoding. All positions with <90% site coverage (Poaceae OMT phylogeny, Figure S1) or < 80% site coverage (phylogeny of the *FOMT2-BX10* clade, Figure 1) were eliminated. Ambiguous bases were allowed at any position. To identify the best-fitting nucleotide substitution model for each dataset, a substitution model test was performed with MEGA7 (for substitution model used, see respective figure legends). A bootstrap resampling analysis with 1000 replicates was performed to evaluate the topology of the *FOMT2*-like subtree. Amino acid sequence alignments were generated with MEGA 7 and visualized with BioEdit.

### 4.9. Homology Modeling and Molecular Docking

A homology model of ZmFOMT2 was generated using the Swiss Model server (https://swissmodel.expasy.org/; accessed on 28 April 2021 [50]) based on isoflavone 4′-*O*-methyltransferase from *M. truncatula* (PDB-ID: 1ZG3; [42]). Docking of naringenin into the homology model of ZmFOMT2 was performed using AutoDock Vina (http://vina.scripps.edu/; accessed on 2 August 2021 [51]) with the grid box (size x/y/z = 26 Å) centered on His271 (C-2) of the catalytic triad and with the exhaustiveness set to 8. AutoDock Tools 1.5.7 (https://ccsb.scripps.edu/mgltools/ accessed on 2 August 2021) was used to prepare protein and ligand files for use in AutoDock Vina. Visualization was performed with PyMOL 0.99rc6 (https://pymol.org/2/ accessed on 4 August 2021). All binding modes of naringenin in the active site pocket (Table S2) were used in combination to represent the 6 Å area shown in Figure 3b.

### 4.10. Accession Numbers

Sequence data for the genes amplified and cloned in this study can be found in the NCBI GenBank (https://www.ncbi.nlm.nih.gov/genbank/ accessed on 13 December 2021) under the following identifiers: *SbFOMT2* (OL907152) and *Pvir2300-like* (OL907153).

## Data Availability

Data are contained within the article and Supplementary Materials.

## Supplementary Materials

The following are available online, Figure S1: Phylogenetic tree of Poaceae OMT genes similar to *ZmFOMT2*; Figure S2: Enzymatic activity of *FOMT2*-like group members with different flavonoid substrates; Figure S3: BX OMTs catalyze the 3-*O*-methylation of flavonols; Figure S4: Amino acid sequence alignment of ZmFOMT2 and ZmBX10 with isoflavone OMTs; Figure S5: Amino acid sequence alignment of OMTs in the *FOMT2*-like group; Figure S6: Codon-optimized gene sequences of Poaceae OMTs synthesized for expression in *E. coli*; Table S1: Enzymatic activity of Poaceae OMTs in the *FOMT2*-like group with different substrates; Table S2: Resulting binding modes for docking of naringenin into the homology model of ZmFOMT2 using AutoDock Vina (http://vina.scripps.edu/; Trott and Olson, 2010) with the grid box (size x/y/z = 26 Å) centered on His271 (C-2) base of the catalytic triad and with the exhaustiveness set to 8; Table S3: Other possible mutation sites; Table S4: PCR primers for the amplification of full-length ORFs of investigated OMTs and for site-directed mutagenesis; Table S5: MS settings used for the analysis on the QTRAP 6500+; Table S6: Mass analyzer settings used for the analysis of BXs and flavonoids on the QTRAP 6500+; Table S7: Phytozome 13 (https://phytozome-next.jgi.doe.gov/) datasets used and corresponding references for the phylogenetic analysis shown in Figure 1 and Figure S1.
