# Supplementary material for: Evolution of DIMBOA-Glc O-Methyltransferases from Flavonoid O-Methyltransferases in the Grasses
Source: Molecules. 2022 Feb 2;27(3):1007. doi: 10.3390/molecules27031007 (PMC8839659; doi:10.3390/molecules27031007)
Supplement: Supplementary file 1 [file molecules-27-01007-s001.zip › 2022-01-20_supplemental_tables_combined.pdf]

Table S1. Enzymatic activity of Poaceae OMTs in the *FOMT2*-like group with different substrates. Data belong to the experiment shown in Figure 2 and Figure S2 (panel b and c). The purified recombinant enzymes as well as an empty vector control (EV) were incubated with the potential substrates listed in presence of the cosubstrate SAM. Reaction products were analyzed by LC-MS/MS. Product formation is given as mean area ± SE (n = 3). Numbers in grey are considered as trace activities, because they represent product formation rates less than 1% relative to the most active enzyme, which is either ZmBX12 or ZmFOMT2. Abbreviations: Me, methyl; nd, not detected.

Substrate	Analyte	Mean area ± SE									
		EV	ShFOMT2	SbFOMT2	ZmFOMT2	ZmBX14	ZmBX12	ZnGBZQ	ZnBX10	Pvag9400	Pv2300-like
DIMBOA-Glc	HDMBOA-Glc	nd	nd	nd	nd	2.3E+04	1.8E+05	nd	4.1E+04	nd	nd
		-	-	-	-	± 7.6E+02	± 8.7E+03	-	± 2.2E+03	-	-
Naringenin	5-*O*-Me-Naringenin	nd	1.9E+06	1.1E+07	6.5E+07	5.5E+03	3.9E+04	8.3E+03	4.3E+03	3.4E+04	nd
		-	± 5.6E+04	± 2.1E+05	± 1.0E+06	1.0E+03	± 2.7E+02	4.6E+02	4.6E+02	± 2.8E+03	-
2-OH-Naringenin	*O*-Me-2-OH-Naringenin_P1	nd	7.9E+05	8.6E+06	1.1E+07	nd	nd	nd	nd	3.1E+03	-
		-	± 1.5E+04	± 1.8E+05	± 2.8E+05	-	-	-	-	± 4.4E+02	-
	O-Me-2-OH-Naringenin_P2	nd	3.9E+04	5.1E+05	6.5E+05	nd	nd	nd	nd	nd	-
		-	± 1.2E+03	± 3.4E+04	± 3.6E+03	-	-	-	-	-	-
	Xilonenin (keto)	nd	nd	4.3E+05	1.7E+06	nd	nd	nd	nd	nd	-
		-	-	± 2.7E+04	± 1.1E+05	-	-	-	-	-	-
	Xilonenin (enol)	nd	nd	1.1E+06	4.6E+06	nd	nd	nd	nd	nd	-
		-	-	± 2.8E+04	± 1.3E+05	-	-	-	-	-	-
Apigenin	5-*O*-Me-Apigenin	nd	1.1E+06	4.2E+06	5.2E+07	nd	1.5E+05	nd	nd	1.5E+05	-
		-	± 1.5E+04	± 3.3E+05	± 3.8E+05	-	± 1.3E+02	-	-	± 2.6E+03	-
Scutellarein	5-*O*-Me-Scutellarein	nd	4.8E+05	3.2E+06	1.9E+07	1.3E+04	1.8E+04	nd	nd	8.7E+03	nd
		-	± 8.3E+03	± 2.8E+04	± 6.2E+04	± 3.3E+03	± 2.0E+02	-	-	± 9.8E+02	-
Dihydrokaemfperol	5-*O*-Me-Dihydrokaempferol	nd	2.6E+04	3.2E+05	2.7E+06	nd	nd	nd	nd	nd	-
		-	± 7.5E+02	± 8.5E+02	± 7.4E+04	-	-	-	-	-	-
Kaempferol	5-*O*-Me-Kaempferol	nd	7.2E+05	4.1E+06	4.3E+07	6.0E+04	2.0E+04	nd	nd	2.1E+04	nd
		-	± 3.8E+04	± 7.6E+04	± 5.9E+05	± 2.0E+04	± 3.0E+02	-	-	± 3.3E+03	-
	Isokaempferide	nd	nd	9.8E+04	1.7E+04	nd	5.8E+06	nd	3.9E+05	nd	nd
		-	-	± 2.3E+03	± 2.5E+03	-	± 6.6E+04	-	± 1.1E+04	-	-
Quercetin	5-*O*-Me-Quercetin	nd	3.9E+05	1.1E+05	3.6E+07	4.6E+04	nd	nd	nd	6.9E+03	-
		-	± 2.3E+04	± 5.3E+03	± 9.2E+05	± 2.0E+04	-	-	-	± 7.1E+02	-

Table S2. Resulting binding modes for docking of naringenin into the homology model of ZmFOMT2 using AutoDock Vina (http://vina.scripps.edu/; Trott and Olson, 2010) with the grid box (size x/y/z = 26 Å) centered on His271 (C-2) base of the catalytic triad and with the exhaustiveness set to 8. The binding mode displayed in Figures 3a and 3b is highlighted in grey.

Mode #	Binding affinity (kcal/mol)	Distance from best mode (RMSD lower bound)	Distance from best mode (RMSD upper bound)
1	-7.0	0.000	0.000
2	-6.5	6.297	9.188
3	-6.5	3.298	8.730
4	-6.5	1.240	2.743
5	-6.4	2.578	8.358
6	-6.3	3.695	6.598
7	-6.3	6.088	9.199
8	-6.2	2.337	3.544
9	-6.2	3.132	5.571

Table S3. Other possible mutation sites. Listed are amino acid residues that differ between most, but not all, functional ZmFOMT2-like FOMTs and BX OMTs. The residues are also not restricted to the putative active site. Amino acids present in ZmFOMT2 and ZmBX10 are highlighted in bold.

Alignment position (Figure S5)	ZmFOMT2 sequence position	Amino acid in FOMTs (ZmFOMT2/3 / SbFOMT2 / ShFOMT2)	Amino acid in BX OMTs (ZmBX10/11/12 / ZnBX10)
175	130	**H**	**Q**
179	134	**V** / Q	**A**
265	199	**V**	**T** / M
338	268	**T**	**K**
381	308	**G** / D	**K**
396	315	**L**	**Q**
444	/	**-**	**I**

Table S4. PCR primers for the amplification of full-length ORFs of investigated OMTs and for site-directed mutagenesis.

Primer name	Target gene (GenBank accession)	Primer Sequence (5' → 3')	Restriction recognition site / mutation	Application
Sb-001G354400-F	*Sb001G354400*	CACCATGGCACCCGCGGAGGAGAG		TOPO cloning
Sb-001G354400-R		TCATGGATAGACCTCGATGAC		
Pv-9NG562300-IBA-F	*Pvir9NG562300*	ATGGTAGGTCTCAGCGCATGGCATCATCATCGCTCCCTG	*BsaI*	pASK-IBA37plus cloning
Pv-9NG562300-IBA-R		ATGGTAGGTCTCATATCAAGGGTAGAGTTCGATGATTGACAT		
Fomt2-Mut8+9-F2	*ZmFOMT2* (MZ484743)	CGACCTCTTGCACCATTCCCTGTGCTACGCCAAATCGCTC	W16L + Q18H	site-directed mutagenesis
Fomt2-Mut8+9-R2		CACAGGGAATGGTGCAAGAGGTCGACGTGAGCTTCGAGC		
Fomt2-W22-Mut3-F		GGTAATAATCTTGGACGTGGTAGTTGGATATGG	M303V	
Fomt2-W22-Mut3-R		CCATATCCAACTACCACGTCCAAGATTATTACC		
Fomt2-W22-Mut1+2-F2		GTTATGTTTGATTTGTATATGATGGCGGTTAATGGAGCTGAGCGC	I325M + T327A	
Fomt2-W22-Mut1+2-R2		GCGCTCAGCTCCATTAACCGCCATCATATACAAATCAAACATAAC		
Fomt2-W22-Mut4+5-F2		CAAAATCCTACCAATCCTTGGTGACGTATCAGTCATCGAGGTCTATCC	A358D + L359V	
Fomt2-W22-Mut4+5-R2		GGATAGACCTCGATGACTGATACGTCACCAAGGATTGGTAGGATTTTG		
Fomt2-W22-Mut1+2+6-F		GTATATGATGGCGGTTAATGGAGTTGAGCGCGACGAGCAAGAGTGG	I325M + T327A + A331V	
Fomt2-W22-Mut1+2+6-R		CCACTCTTGCTCGTCGCGCTCAACTCCATTAACCGCCATCATATAC		
Fomt2-W22-Mut4+5+7-F		GACTACAAAATCCTACCAATCATTGGTGACGTATCAGTCATCG	A358D + L359V + L356I	
Fomt2-W22-Mut4+5+7-R		CGATGACTGATACGTCACCAATGATTGGTAGGATTTTGTAGTC		

Table S5. MS settings used for the analysis on the QTRAP 6500+.

**Turbospray ESI ion source settings**		
Analytes	**flavonoids**	**benzoxazinoids**
Ionization mode:	positive	negative
Ion spray voltage:	+5500 V	-4500 V
Turbo gas temperature:	650°C	650°C
Curtain gas:	40 psi	40 psi
Collision gas:	medium level	medium level
Nebulizer gas:	60 psi	70 psi
Heating gas:	60 psi	70 psi

Table S6. Mass analyzer settings used for the analysis of BXs and flavonoids on the QTRAP 6500+. Retention time (RT) was used to distinguish between compounds with the same MRM transition. Compounds marked with (*) were verified by an authentic standard, while the remaining *O*-methylflavonoids were inferred from the specific ZmFOMT2 enzymatic activity as described previously in Förster et al. (2021). Abbreviations: Me, methyl; DP, declustering potential; EP, entrance potential; CE, collision energy; CXP, collision cell exit potential; V, volts.

**Compound**	**RT (min)**	**MRM transition *m/z***	**DP (V)**	**EP (V)**	**CE (V)**	**CXP (V)**
**Benzoxazinoids (negative mode)**						
DIMBOA-Glc*	4.35	372 $[M-H]^-$ → 210	-40	-4	-15	-4
HDMBOA-Glc*	5.16	432 $[M+FA-H]^-$ → 356	-40	-4	-20	-3
**Flavonoids (positive mode)**						
Apigenin*	6.59	271 $[M+H]^+$ → 153	50	3.5	41	4
Naringenin*	6.64	273 $[M+H]^+$ → 153	41	4.5	31	4
5-*O*-Me-Apigenin*	4.94	285 $[M+H]^+$ → 270	50	6	31	8
5-*O*-Me-Naringenin*	4.98	287 $[M+H]^+$ → 167	36	5	31	6
Scutellarein*	5.32	287 $[M+H]^+$ → 123	50	3.5	47	4
Kaempferol*	6.80	287 $[M+H]^+$ → 153	50	2	39	4
Dihydrokaempferol*	5.11	289 $[M+H]^+$ → 243	50	4	15	6
2-Hydroxynaringenin*	5.52	289 $[M+H]^+$ → 121	21	5	21	14
5-*O*-Me-Scutellarein*	4.67	301 $[M+H]^+$ → 286	50	3	33	6
5-*O*-Me-Kaempferol	5.50	301 $[M+H]^+$ → 286	50	2	35	6
3-*O*-Me-Kaempferol (Isokaempferide)*	7.03	301 $[M+H]^+$ → 286	50	4	27	8
5-*O*-Me-Dihydrokaempferol	4.40	303 $[M+H]^+$ → 107	50	4	55	2
Quercetin*	5.93	303 $[M+H]^+$ → 153	50	7	45	4
O-Me-2-Hydroxynaringenin (peak 1)	6.41	303 $[M+H]^+$ → 167	21	5	21	20
O-Me-2-Hydroxynaringenin (peak 2)	8.29	303 $[M+H]^+$ → 167	21	5	21	20
5-*O*-Me-Quercetin	4.78	317 $[M+H]^+$ → 302	50	7	35	6
O-DiMe-2-Hydroxynaringenin (Xilonenin; peak 1)*	5.48	317 $[M+H]^+$ → 181	1	5	21	20
O-DiMe-2-Hydroxynaringenin (Xilonenin; peak 2)*	6.78	317 $[M+H]^+$ → 181	1	5	21	20

Table S7. Phytozome 13 (https://phytozome-next.jgi.doe.gov/) data sets used and corresponding references for the phylogenetic analysis shown in Figure 1 and Figure S1.

Plant species	Data set version	Reference
Brachipodium distachyon	3.1	https://phytozome-next.jgi.doe.gov/info/Bdistachyon_v3_1
Brachipodium stacei	1.1	Gordon et al., 2020
Eleusine coracana	1.1	https://phytozome-next.jgi.doe.gov/info/Ecoracana_v1_1
Hordeum vulgare	v1	Beier et al., 2017
Miscanthus sinensis	7.1	Mitros et al., 2020
Oropetium thomaeum	1.0	VanBuren et al., 2015
Oryza sativa	7.0	Ouyang et al., 2007
Panicum hallii	3.2	Lovell et al., 2018
Panicum hallii HAL	2.2	Lovell et al., 2018
Panicum virgatum	5.1	Lovell et al., 2021
Paspalum vaginatum	3.1	DOE-JGI, https://phytozome-next.jgi.doe.gov/info/Pvaginatum_v3_1
Setaria italica	2.2	Bennetzen et al., 2012
Setaria viridis	2.1	Mamidi et al., 2020
Sorghum bicolor	3.1.1	McCormick et al., 2018
Sorghum bicolor Rio	2.1	Cooper et al., 2019
Triticum aestivum	2.2	International Wheat Genome Sequencing Consortium (IWGSC), 2014
Urochloa fusca	1.1	DOE-JGI, https://phytozome-next.jgi.doe.gov/info/Ufusca_v1_1
Zea mays	B73_v4	Jiao et al., 2017

Beier, S., Himmelbach, A., Colmsee, C., Zhang, X.Q., Barrero, R.A., Zhang, Q., Li, L., Bayer, M., Bolser, D., Taudien, S., Groth, M., Felder, M., Hastie, A., Simkova, H., Stankova, H., Vrana, J., Chan, S., Munoz-Amatriain, M., Ounit, R., Wanamaker, S., Schmutzer, T., Aliyeva-Schnorr, L., Grasso, S., Tanskanen, J., Sampath, D., Heavens, D., Cao, S., Chapman, B., Dai, F., Han, Y., Li, H., Li, X., Lin, C., McCooke, J.K., Tan, C., Wang, S., Yin, S., Zhou, G., Poland, J.A., Bellgard, M.I., Houben, A., Dolezel, J., Ayling, S., Lonardi, S., Langridge, P., Muehlbauer, G.J., Kersey, P., Clark, M.D., Caccamo, M., Schulman, A.H., Platzer, M., Close, T.J., Hansson, M., Zhang, G., Braumann, I., Li, C., Waugh, R., Scholz, U., Stein, N., and Mascher, M. (2017). Construction of a map-based reference genome sequence for barley, *Hordeum vulgare L*. Sci Data **4,** 170044.

Bennetzen, J.L., Schmutz, J., Wang, H., Percifield, R., Hawkins, J., Pontaroli, A.C., Estep, M., Feng, L., Vaughn, J.N., Grimwood, J., Jenkins, J., Barry, K., Lindquist, E., Hellsten, U., Deshpande, S., Wang, X., Wu, X., Mitros, T., Triplett, J., Yang, X., Ye, C.Y., Mauro-Herrera, M., Wang, L., Li, P., Sharma, M., Sharma, R., Ronald, P.C., Panaud, O., Kellogg, E.A., Brutnell, T.P., Doust, A.N., Tuskan, G.A., Rokhsar, D., and Devos, K.M. (2012). Reference genome sequence of the model plant *Setaria*. Nat Biotechnol **30,** 555-561.

Cooper, E.A., Brenton, Z.W., Flinn, B.S., Jenkins, J., Shu, S., Flowers, D., Luo, F., Wang, Y., Xia, P., Barry, K., Daum, C., Lipzen, A., Yoshinaga, Y., Schmutz, J., Saski, C., Vermerris, W., and Kresovich, S. (2019). A new reference genome for *Sorghum bicolor* reveals high levels of sequence similarity between sweet and grain genotypes: implications for the genetics of sugar metabolism. Bmc Genomics **20,** 420.

Gordon, S.P., Contreras-Moreira, B., Levy, J.J., Djamei, A., Czedik-Eysenberg, A., Tartaglio, V.S., Session, A., Martin, J., Cartwright, A., Katz, A., Singan, V.R., Goltsman, E., Barry, K., Dinh-Thi, V.H., Chalhoub, B., Diaz-Perez, A., Sancho, R., Lusinska, J., Wolny, E., Nibau, C., Doonan, J.H., Mur, L.A.J., Plott, C., Jenkins, J., Hazen, S.P., Lee, S.J., Shu, S., Goodstein, D., Rokhsar, D., Schmutz, J., Hasterok, R., Catalan, P., and Vogel, J.P. (2020). Gradual polyploid genome evolution revealed by pan-genomic analysis of *Brachypodium hybridum* and its diploid progenitors. Nat Commun **11,** 3670.

IWGSC. (2014). A chromosome-based draft sequence of the hexaploid bread wheat (*Triticum aestivum*) genome. Science **345,** 1251788.

Jiao, Y., Peluso, P., Shi, J., Liang, T., Stitzer, M.C., Wang, B., Campbell, M.S., Stein, J.C., Wei, X., Chin, C.S., Guill, K., Regulski, M., Kumari, S., Olson, A., Gent, J., Schneider, K.L., Wolfgruber, T.K., May, M.R., Springer, N.M., Antoniou, E., McCombie, W.R., Presting, G.G., McMullen, M., Ross-Ibarra, J., Dawe, R.K., Hastie, A., Rank, D.R., and Ware, D. (2017). Improved maize reference genome with single-molecule technologies. Nature **546,** 524-527.

Lovell, J.T., Jenkins, J., Lowry, D.B., Mamidi, S., Sreedasyam, A., Weng, X., Barry, K., Bonnette, J., Campitelli, B., Daum, C., Gordon, S.P., Gould, B.A., Khasanova, A., Lipzen, A., MacQueen, A., Palacio-Mejia, J.D., Plott, C., Shakirov, E.V., Shu, S., Yoshinaga, Y., Zane, M., Kudrna, D., Talag, J.D., Rokhsar, D., Grimwood, J., Schmutz, J., and Juenger, T.E. (2018). The genomic landscape of molecular responses to natural drought stress in *Panicum hallii*. Nat Commun **9,** 5213.

Lovell, J.T., MacQueen, A.H., Mamidi, S., Bonnette, J., Jenkins, J., Napier, J.D., Sreedasyam, A., Healey, A., Session, A., Shu, S., Barry, K., Bonos, S., Boston, L., Daum, C., Deshpande, S., Ewing, A., Grabowski, P.P., Haque, T., Harrison, M., Jiang, J., Kudrna, D., Lipzen, A., Pendergast, T.H.t., Plott, C., Qi, P., Saski, C.A., Shakirov, E.V., Sims, D., Sharma, M., Sharma, R., Stewart, A., Singan, V.R., Tang, Y., Thibivillier, S., Webber, J., Weng, X., Williams, M., Wu, G.A., Yoshinaga, Y., Zane, M., Zhang, L., Zhang, J., Behrman, K.D., Boe, A.R., Fay, P.A., Fritschi, F.B., Jastrow, J.D., Lloyd-Reilley, J., Martinez-Reyna, J.M., Matamala, R., Mitchell, R.B., Rouquette, F.M., Jr., Ronald, P., Saha, M., Tobias, C.M., Udvardi, M., Wing, R.A., Wu, Y., Bartley, L.E., Casler, M., Devos, K.M., Lowry, D.B., Rokhsar, D.S., Grimwood, J., Juenger, T.E., and Schmutz, J. (2021). Genomic mechanisms of climate adaptation in polyploid bioenergy switchgrass. Nature **590,** 438-444.

Mamidi, S., Healey, A., Huang, P., Grimwood, J., Jenkins, J., Barry, K., Sreedasyam, A., Shu, S., Lovell, J.T., Feldman, M., Wu, J., Yu, Y., Chen, C., Johnson, J., Sakakibara, H., Kiba, T., Sakurai, T., Tavares, R., Nusinow, D.A., Baxter, I., Schmutz, J., Brutnell, T.P., and Kellogg, E.A. (2020). A genome resource for green millet *Setaria viridis* enables discovery of agronomically valuable loci. Nat Biotechnol **38,** 1203-1210.

McCormick, R.F., Truong, S.K., Sreedasyam, A., Jenkins, J., Shu, S., Sims, D., Kennedy, M., Amirebrahimi, M., Weers, B.D., McKinley, B., Mattison, A., Morishige, D.T., Grimwood, J., Schmutz, J., and Mullet, J.E. (2018). The *Sorghum bicolor* reference genome: improved assembly, gene annotations, a transcriptome atlas, and signatures of genome organization. Plant J **93,** 338-354.

Mitros, T., Session, A.M., James, B.T., Wu, G.A., Belaffif, M.B., Clark, L.V., Shu, S., Dong, H., Barling, A., Holmes, J.R., Mattick, J.E., Bredeson, J.V., Liu, S., Farrar, K., Glowacka, K., Jezowski, S., Barry, K., Chae, W.B., Juvik, J.A., Gifford, J., Oladeinde, A., Yamada, T., Grimwood, J., Putnam, N.H., De Vega, J., Barth, S., Klaas, M., Hodkinson, T., Li, L., Jin, X., Peng, J., Yu, C.Y., Heo, K., Yoo, J.H., Ghimire, B.K., Donnison, I.S., Schmutz, J., Hudson, M.E., Sacks, E.J., Moose, S.P., Swaminathan, K., and Rokhsar, D.S. (2020). Genome biology of the paleotetraploid perennial biomass crop *Miscanthus*. Nat Commun **11,** 5442.

Ouyang, S., Zhu, W., Hamilton, J., Lin, H., Campbell, M., Childs, K., Thibaud-Nissen, F., Malek, R.L., Lee, Y., Zheng, L., Orvis, J., Haas, B., Wortman, J., and Buell, C.R. (2007). The TIGR Rice Genome Annotation Resource: improvements and new features. Nucleic Acids Res **35,** D883-887.

VanBuren, R., Bryant, D., Edger, P.P., Tang, H., Burgess, D., Challabathula, D., Spittle, K., Hall, R., Gu, J., Lyons, E., Freeling, M., Bartels, D., Ten Hallers, B., Hastie, A., Michael, T.P., and Mockler, T.C. (2015). Single-molecule sequencing of the desiccation-tolerant grass *Oropetium thomaeum*. Nature **527,** 508-511.
