# Supplementary material for: Evolution of DIMBOA-Glc O-Methyltransferases from Flavonoid O-Methyltransferases in the Grasses
Source: Molecules. 2022 Feb 2;27(3):1007. doi: 10.3390/molecules27031007 (PMC8839659; doi:10.3390/molecules27031007)
Supplement: Supplementary file 1 [file molecules-27-01007-s001.zip › 2022-01-21_supplemental_figures.pdf]

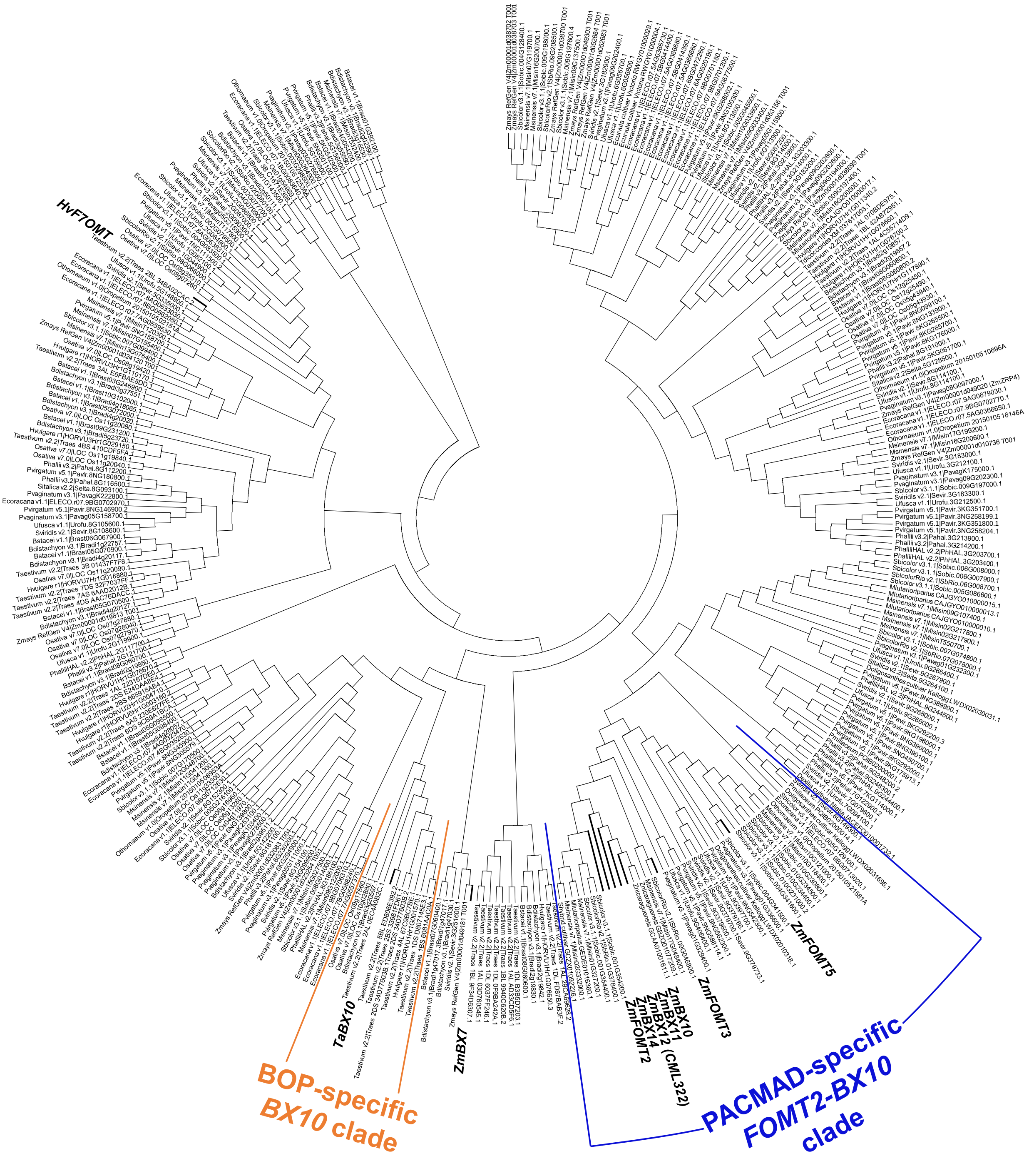

Figure S1. Phylogenetic tree of Poaceae OMT genes similar to *ZmFOMT2.* OMTs were identified by BLASTP analysis with ZmFOMT2 as query and using Poaceae protein datasets available in the Phytozome 13 (https://phytozome-next.jgi.doe.gov/) and NCBI (https://www.ncbi.nlm.nih.gov/) databases. Additional sequences were obtained from the NCBI TSA database (https://www.ncbi.nlm.nih.gov/genbank/tsa/) by a local BLAST search using BioEdit followed by an NCBI search for full sequences. Only genes (ORFs) with ≥ 80% query coverage and a corresponding amino acid identity of ≥ 40% were included. The tree was inferred using the maximum likelihood method based on the General Time Reversible model, including gamma distributed rate variation among sites (+G, 1.8577). All positions with < 90% site coverage were eliminated. *OMTs* previously characterized in the literature are highlighted in black bold, and the "PACMAD-specific *FOMT2-BX10* clade" and "BOP-specific *BX10* clade" are marked in blue and orange, respectively. Sequences of the following species were included in the tree: *Brachypodium distachyon*; *Brachypodium stacei*; *Dichanthelium oligosanthes* Kellogg; *Digitaria exilis* Niatia; *Eleusine coracana*; *Eragrostis curvula* Victoria; *Hordeum vulgare* (*Hv*); *Miscanthus lutarioriparius*; *Miscanthus sinensis*; *Oropetium thomaeum*; *Oryza sativa*; *Panicum hallii*; *Panicum hallii* HAL; *Panicum miliaceum*; *Panicum virgatum*; *Paspalum vaginatum*; *Saccharum* hybrid; *Setaria italica*; *Setaria viridis*; *Sorghum bicolor*; *Sorghum bicolor* Rio; *Triticum aestivum* (*Ta*); *Triticum dicoccoides*; *Urochloa fusca*; *Zea mays* (*Zm*); *Zea nicaraguensis*.

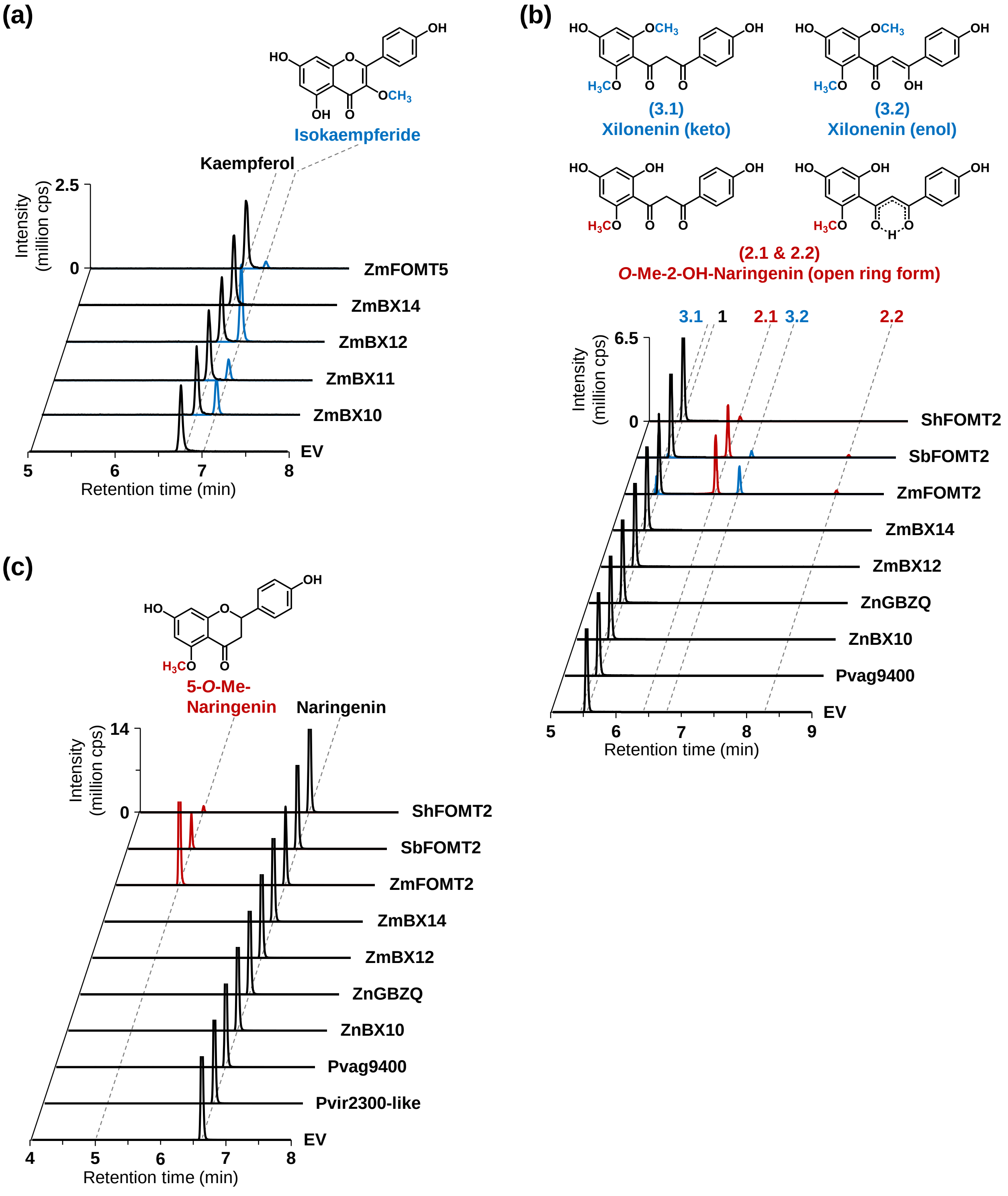

Figure S2. Enzymatic activity of *FOMT2*-like group members with different flavonoid substrates. The purified recombinant enzymes as well as an empty vector control (EV) were incubated with the potential substrates kaempferol (**a**), 2-hydroxynaringenin (**b**), and naringenin (**c**) in presence of the cosubstrate SAM. Reaction products were analyzed by LC-MS/MS. Chromatograms of specific MRM transitions (see methods section) are shown. The upper part of each panel shows the structures of the enzyme products, with the attached methyl groups highlighted in blue or red. All assays were performed in technical triplicates. Peak numbers in panel (b): 1, 2-hydroxynaringenin; 2.1 and 2.2, *O*-methyl-2-hydroxynaringenin; 3.1, xilonenin (keto); 3.2, xilonenin (enol). Abbreviations: Me, methyl; cps, counts per second. **Note:** The data shown in panel (a) were collected independently from the experiments shown in Figure 2 and panel (b) and (c).

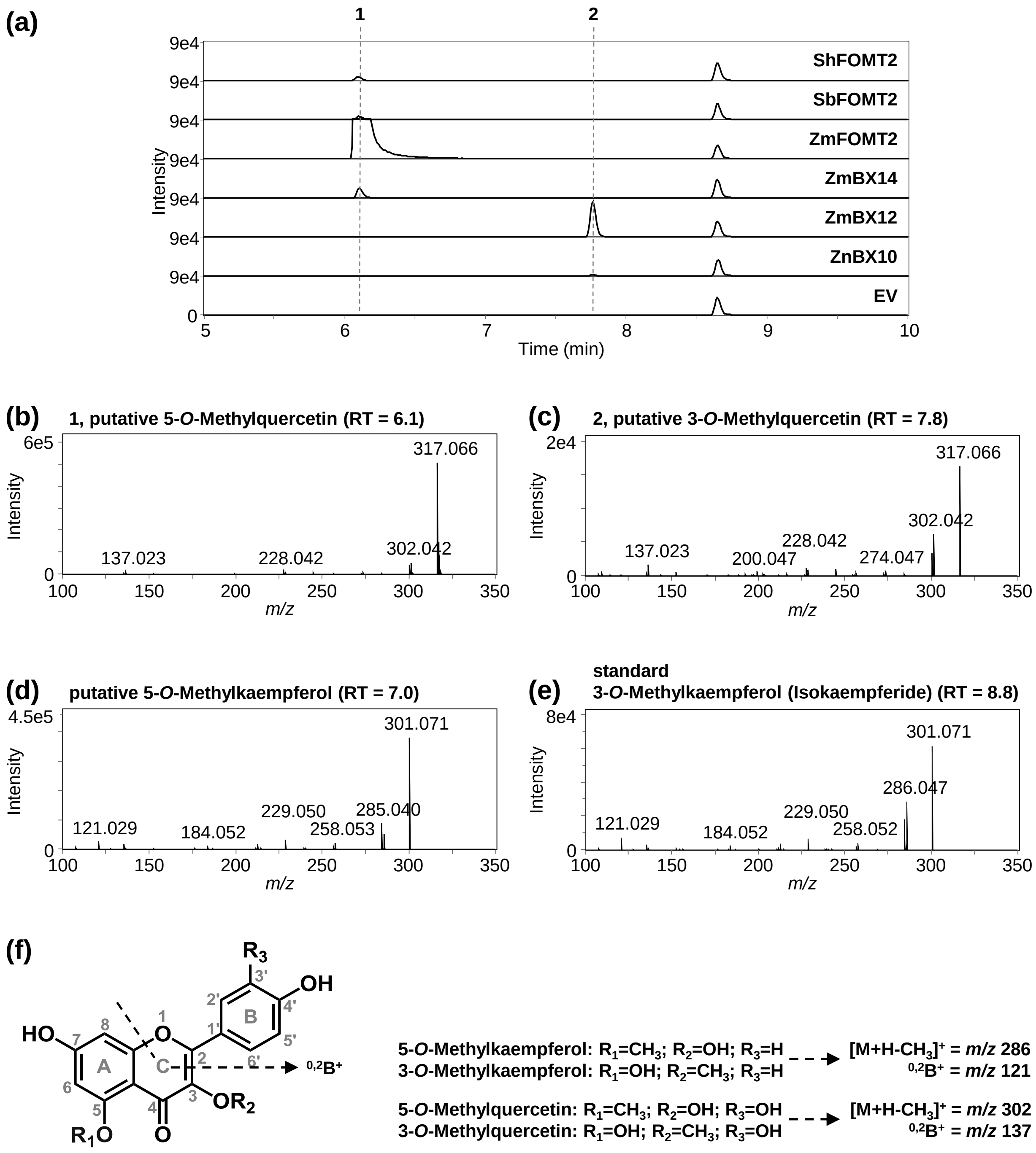

Figure S3. BX OMTs catalyze the 3-*O*-methylation of flavonols. Extracted ion chromatograms (*m/z* 317.066 $[M+H]^+$) (**a**) and MS/MS spectra (**b-c**) of quercetin products produced by *FOMT2*-like FOMTs and BX OMTs. For comparison the MS/MS spectra of the corresponding kaempferol products putative 5-*O*-methylkaempferol and 3-*O*-methylkaempferol (isokaempferide) are shown (**d-e**). Fragmentation patterns are similar and consistent with mono-*O*-methylation on the flavonol A- or C-ring, as a typical B-ring fragment is observed (**f**). The position of *O*-methylation can be distinguished by retention time (RT). Enzyme assays using quercetin or kaempferol as substrate were analyzed using untargeted LC-MS (full scan and auto MS/MS mode) as described in the methods section. The MS/MS spectrum of isokaempferide was obtained using the purified compound. 1, putative 5-*O*-methylquercetin; 2, putative 3-*O*-methylquercetin.

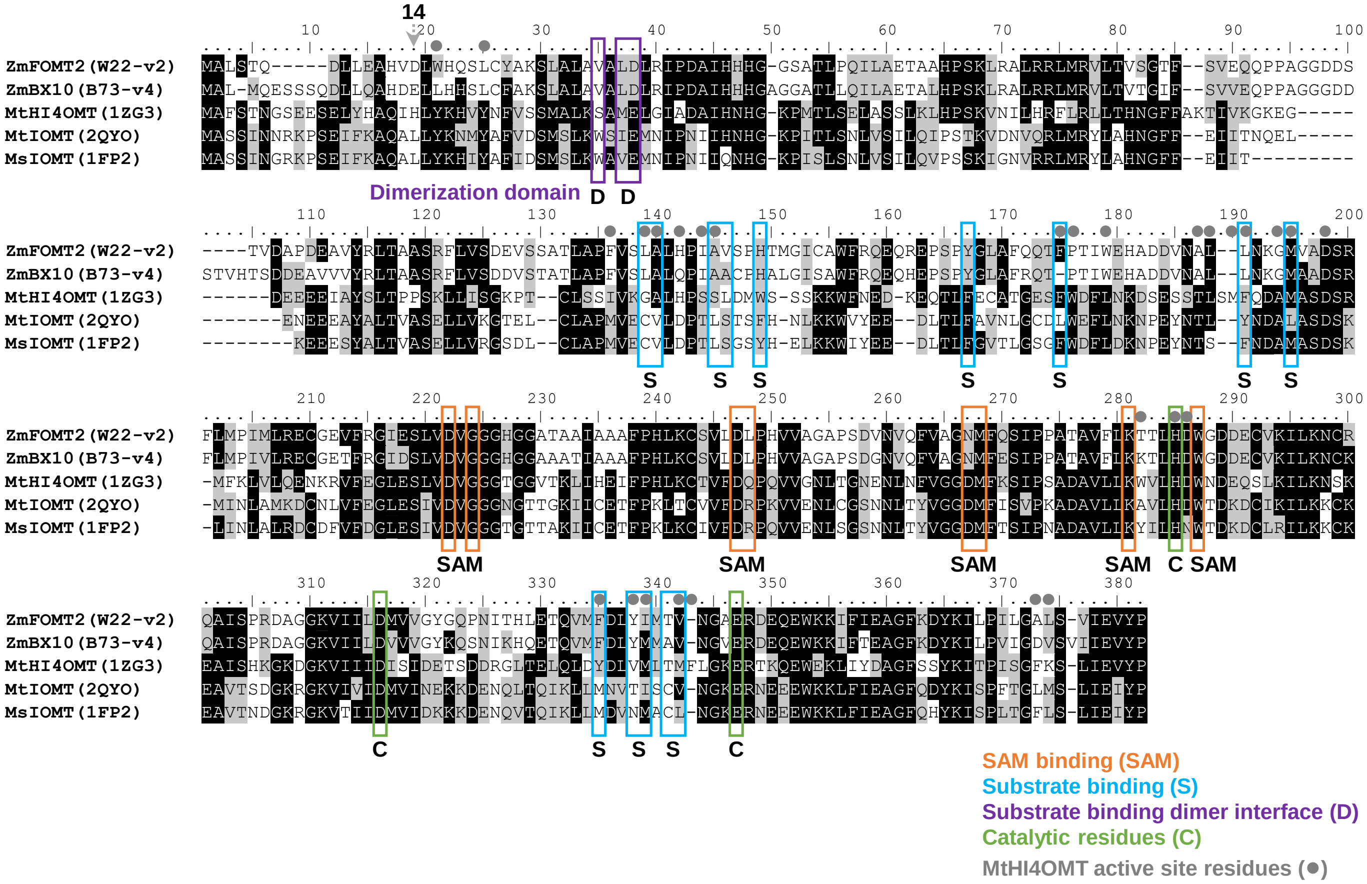

Figure S4. Amino acid sequence alignment of ZmFOMT2 and ZmBX10 with isoflavone OMTs. Isoflavone 4'-OMT from *Medicago truncatula* (MtHI4OMT; PDB-ID: 1ZG3), Isoflavone-OMT from *M. truncatula* (MtIOMT; PDB-ID: 2QYO), and Isoflavone-OMT from *Medicago sativa* (MsIOMT; PDB-ID: 1FP2) were identified as the best templates for homology modelling with ZmFOMT2 (MZ484743) using the Swiss-Model server (https://swissmodel.expasy.org/). The sequences were aligned using MEGA7 and visualized with BioEdit. Identical amino acids are shaded in black and similar amino acids in grey. Amino acid residues involved in SAM binding (orange), substrate binding (blue), substrate binding from the second polypeptide chain of the homodimer (purple), catalysis (green) are highlighted (modified from Zubieta et al., 2001). MtHI4OMT active site residues are marked with grey dots (modified from Liu et al., 2006). ZmFOMT2 shares 39%, 34%, 35%, and 84% amino acid identity with MtHI4OMT, MtIOMT, MsIOMT, and ZmBX10, respectively. ZmBX10 is encoded by *Zm00001d029359*. **Note:** Unlike the MaizeGDB database sequence encoded by *Zm00004b033403*/*Zm00004b033399* (W22_v2), the cloned ZmFOMT2 (MZ484743) sequence displayed here contains a D instead of an E in position 14 (grey dashed arrow).

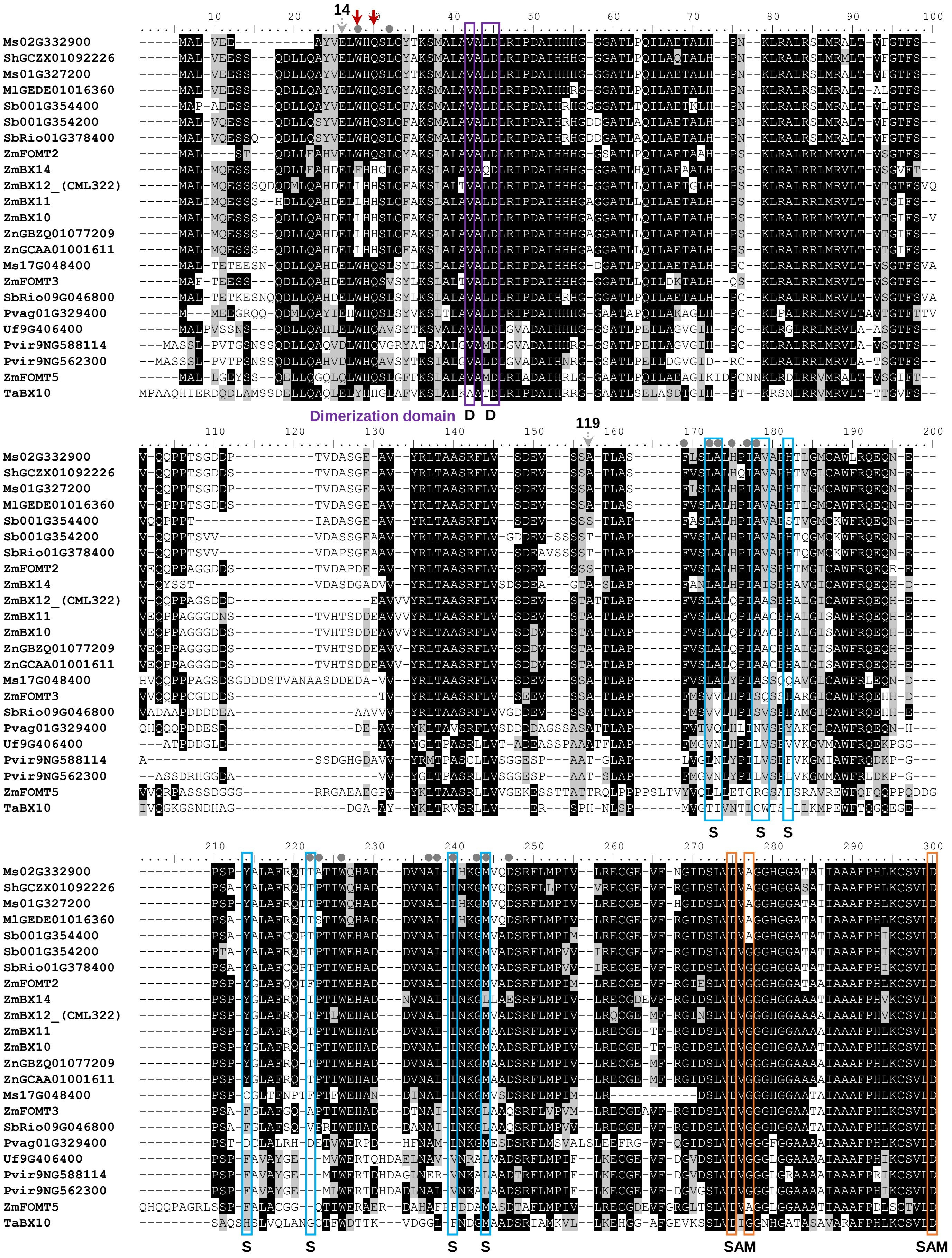
14
119
Ms02G332900
ShGCZX01092226
Ms01G327200
MlGEDE01016360
Sb001G354400
Sb001G354200
SbRio01G378400
ZmFOMT2
ZmBX14
ZmBX12_(CML322)
ZmBX11
ZmBX10
ZnGBZQ01077209
ZnGCAA01001611
Ms17G048400
ZmFOMT3
SbRio09G046800
Pvag01G329400
Uf9G406400
Pvir9NG588114
Pvir9NG562300
ZmFOMT5
TaBX10
Dimerization domain
D D
S S S
S S S S
SAM
SAM

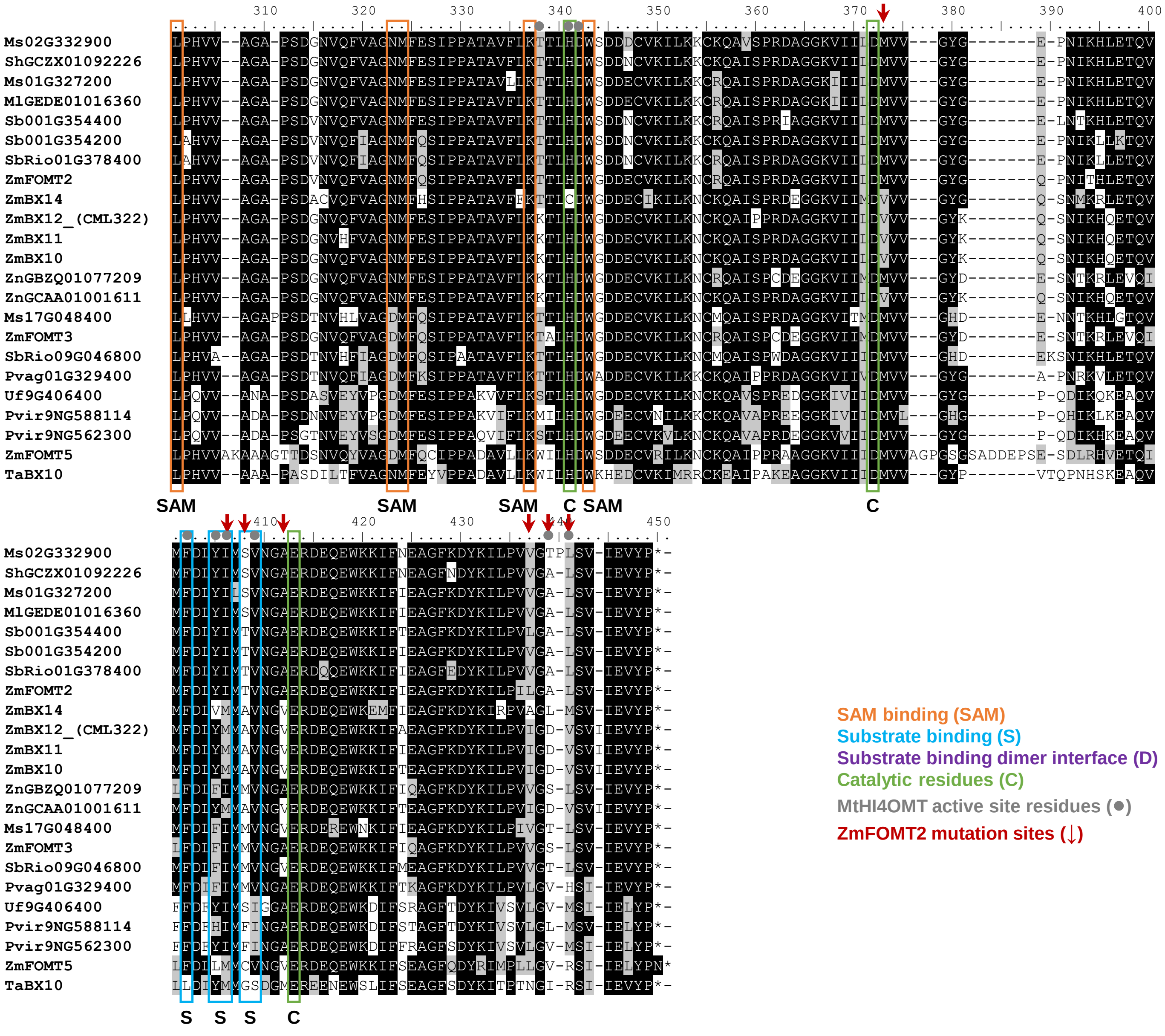

Figure S5. Amino acid sequence alignment of OMTs in the *FOMT2*-like group. The amino acid sequences were aligned using the MUSCLE codon algorithm implemented in MEGA7 and visualized with BioEdit. Identical amino acids are shaded in black and similar amino acids in grey. Amino acid residues involved in SAM binding (orange), substrate binding (blue), substrate binding from the second polypeptide chain of the homodimer (purple), catalysis (green) are highlighted (modified from Zubieta et al., 2001). MtHI4OMT active site residues are marked with grey dots (modified from Liu et al., 2006). ZmFOMT2 mutation sites are marked with red arrows. The amino acid sequences are encoded by *Zm00001d047192* (*ZmFOMT2*), *Zm00001d004921* (*ZmBX14*), *Zm00025ab019610* (*ZmBX12*), *Zm00001d029356* (*ZmBX11*), *Zm00001d029359* (*ZmBX10*), *Zm00001d047194* (*ZmFOMT3*), *Zm00001d051934* (*ZmFOMT5*), and 4AL_C467B516F (*TaBX10*). **Note:** Unlike the cloned W22 sequence displayed in Figure S4, ZmFOMT2 (B73) shown here contains an E instead of a D in position 14 and an A instead of an S in position 119 (grey dashed arrows).

***ShGCZX01092226* codon optimized**

ATGGCACTGGTTGAAGAAAGCAGCCAGGATCTGCTGCAGGCATATGTTGAACTGTGGCATCAGAGCCTGTGTTATGCAAAAAGCATGGCCCTGGC
AGTTGCACTGGATCTGCGTATTCCGGATGCAATTCATCATCATGGTGGTGGTGCAACCCTGCCTCAGATTCTGGCACAGACCGCACTGCATCCGA
ATAAACTGCGTGCACTGCGTAGCCTGATGCGTATGCTGACCGTTTTTGGCACCTTTAGCGTTCAGCAGCCTCCGACCAGCGGTGATGATAGCACC
GTTGATGCAAGCGGTGAAGCAGTTTATCGTCTGACCGCAGCAAGCCGTTTTCTGGTTAGTGATGAAGTTAGCAGCGCAACCCTGGCAAGCTTTGT
TAGCCTGGCGCTGCATCAGATTGCCGTTGCACCGCATACCGTTGGTCTGTGTGCATGGTTTCGTCAAGAACAGAATGAACCGAGCGCATATGCAC
TGGCATTTCGTCAGCCGACACCGACCATTTGGCAGCATGCAGATGATGTTAATGCACTGCTGAATAAAGGTATGGTTCAGGATAGTCGTTTTCTG
CTGCCGATTGTTGTTCGTGAATGTGGTGAAGTTTTTCGCGGTATTGATAGCCTGGTTGATGTTGCCGGTGGTCATGGTGGCGCAAGCGCAATTAT
TGCAGCAGCATTTCCGCATCTGAAATGTAGCGTTCTGGACCTGCCGCATGTTGTTGCGGGTGCACCGAGTGATGGTAATGTTCAGTTTGTTGCAG
GTAACATGTTTGAAAGCATTCCGCCTGCAACCGCAGTTTTTCTGAAAACCACACTGCATGATTGGTCCGATGATAACTGTGTGAAAATCCTGAAA
AAATGCAAGCAGGCAATTAGTCCGCGTGATGCAGGCGGTAAAGTTATTATTCTGGATATGGTTGTTGGTTATGGCGAGCCGAACATTAAACATCT
GGAAACCCAGGTTATGTTCGACCTGTATATTATGAGCGTTAATGGTGCAGAACGTGATGAACAAGAGTGGAAAAAAATCTTTAACGAGGCAGGCT
TCAACGACTACAAAATTCTGCCGGTTGTGGGTGCACTGAGCGTTATTGAAGTTTATCCGTAA

***ZnGBZQ01077209* codon optimized**

ATGGCACTGATGCAAGAAAGCAGCAGCCAGGATCTGCTGCAGGCACATGATGAACTGCTGCATCATAGCCTGTGTTTTGCCAAAAGCCTGGCACT
GGCAGTTGCACTGGATCTGCGTATTCCGGATGCAATTCATCATCATGGTGCCGGTGGTGCAACCCTGCTGCAAATTCTGGCAGAAACCGCACTGC
ATCCGAGCAAACTGCGTGCACTGCGTCGTCTGATGCGTGTTCTGACCGTTACCGGTATTTTTAGCGTTGAACAGCCTCCGGCAGGCGGTGGTGAT
GATAGCACCGTTCATACCTCAGATGATGAAGCCGTTGTTTATCGTCTGACCGCAGCAAGCCGTTTTCTGGTTAGTGATGATGTTAGCACAGCAAC
CCTGGCACCGTTTGTTAGCCTGGCGCTGCAGCCGATTGCAGCATGTCCGCATGCACTGGGTATTAGCGCATGGTTTCGTCAAGAACAGCATGAAC
CGAGTCCGTATGGTCTGGCATTTCGTCAGACCCCGACCATTTGGGAACATGCAGATGATGTGAATGCACTGCTGAATAAAGGTATGGCAGCAGAT
AGTCGTTTTCTGATGCCGATTGTTCTGCGTGAATGTGGTGAAATGTTTCGTGGTATTGATAGCCTGGTTGATGTTGGTGGTGGTCATGGTGGTGC
AGCAGCAGCAATTGCAGCAGCCTTTCCGCATCTGAAATGTAGCGTTCTGGACCTGCCGCATGTTGTTGCGGGTGCACCGAGTGATGGTAATGTTC
AGTTTGTTGCAGGCAACATGTTTGAAAGCATTCCGCCTGCAACCGCAGTTTTTCTGAAAAAAACCCTGCATGATTGGGGTGATGATGAGTGTGTT
AAAATTCTGAAGAATTGCCGTCAGGCAATTAGCCCGTGTGATGAAGGTGGTAAAGTGATTATTATGGATATGGTGGTGGGTTATGATGAGAGCAA
TACCAAACGTCTGGAAGTGCAGATTCTGTTTGACCTGTTTATTATGATGGTGAATGGTGCCGAACGTGATGAACAAGAATGGAAAAAAATCTTTA
TCCAGGCAGGCTTCAAGGACTATAAAATCCTGCCGGTTGTTGGTAGCCTGAGCGTTATTGAAGTTTATCCGTAA

***ZnGCAA01001611* codon optimized**

ATGGCACTGATGCAAGAAAGCAGCAGCCAGGATCTGCTGCAGGCACATGATGAACTGCTGCATCATAGCCTGTGTTTTGCCAAAAGCCTGGCACT
GGCAGTTGCACTGGATCTGCGTATTCCGGATGCAATTCATCATCATGGTGCCGGTGGTGCAACCCTGCTGCAAATTCTGGCAGAAACCGCACTGC
ATCCGAGCAAACTGCGTGCACTGCGTCGTCTGATGCGTGTTCTGACCGTTACCGGTATTTTTAGCGTTGAACAGCCTCCGGCAGGCGGTGGTGAT
GATAGCACCGTTCATACCTCAGATGATGAAGCCGTTGTTTATCGTCTGACCGCAGCAAGCCGTTTTCTGGTTAGTGATGATGTTAGCACAGCAAC
CCTGGCACCGTTTGTTAGCCTGGCGCTGCAGCCGATTGCAGCATGTCCGCATGCACTGGGTATTAGCGCATGGTTTCGTCAAGAACAGCATGAAC
CGAGTCCGTATGGTCTGGCATTTCGTCAGACCCCGACCATTTGGGAACATGCAGATGATGTGAATGCACTGCTGAATAAAGGTATGGCAGCAGAT
AGTCGTTTTCTGATGCCGATTGTTCTGCGTGAATGTGGTGAAATGTTTCGTGGTATTGATAGCCTGGTTGATGTTGGTGGTGGTCATGGTGGTGC
AGCAGCAGCAATTGCAGCAGCCTTTCCGCATCTGAAATGTAGCGTTCTGGACCTGCCGCATGTTGTTGCGGGTGCACCGAGTGATGGTAATGTTC
AGTTTGTTGCAGGCAACATGTTTGAAAGCATTCCGCCTGCAACCGCAGTTTTTCTGAAAAAAACCCTGCATGATTGGGGTGATGATGAGTGTGTT
AAAATTCTGAAGAATTGCAAGCAGGCAATTAGTCCGCGTGATGCAGGCGGTAAAGTTATTATTCTGGATGTTGTTGTGGGCTACAAACAGAGCAA
CATCAAACATCAAGAAACCCAGGTTATGTTCGACCTGTATATGATGGCAGTTAATGGTGTTGAACGTGATGAACAAGAGTGGAAAAAAATCTTTA
CCGAGGCAGGCTTCAAAGACTATAAAATCCTGCCGGTTATTGGTGATGTGAGCGTTATTATTGAAGTGTACCCGTAA

***Pavag01G329400* codon optimized**

ATGATGGAAGAAGGTCGTCAGCAGCAGGATATGCTGCAGGCCTATATTGAACATTGGCATCAGAGCCTGAGCTATGTTAAAAGTCTGACCCTGGC
AGTTGCACTGGATCTGCGTATTCCGGATGCAATTCATCATCATGGTGGTGCAGCAACCGCACCGCAGATTCTGGCAAAAGCAGGTCTGCATCCGT
GTAAACTGCCTGCACTGCGTCGTCTGATGCGTGTTCTGACCGCAGTTACCGGCACCTTTACCACCGTTCAGCACCAGCAGCAGCCGGATGATGAA
AGCGACGATGAAGCAGTGTATAAACTGACAGCAGTTAGCCGTTTTCTGGTGAGTGATGATGACGATGCAGGTAGCAGCGCAAGCGCAACCACACT
GGCACCGTTTGTTACAGTTCAGCTGCATCTGATTAATGTTAGCCCGTATGCAAAAGGTCTGTGTGCATGGTTTCGTCAAGAACAGAATCATCCGA
GCACCGATTGTCTGGCCCTGCGTCATGATGAAACCGTTTGGGAACGTCCGGATCATTTTAATGCAATGCTGAATAAAGGCATGGAAAGCGATAGT
CGTTTTCTGATGTCTGTTGCACTGAGCCTGGAAGAATTTCGTGGTGTTTTTCAGGGTATTGATAGCCTGGTTGATGTTGGTGGTGGTTTTGGTGG
TGCCGCAGCAGCAATTGCAGCAGCATTTCCGCATCTGAAATGTAGCGTTCTGGACCTGCCGCATGTTGTTGCGGGTGCACCGAGCGATACCAATG
TGCAGTTTATTGCCGGTGATATGTTTAAAAGCATTCCGCCTGCAACCGCAGTTTTTCTGAAAACAACCCTGCATGATTGGGCAGATGATGAGTGT
GTGAAAATTCTGAAAAAGTGCAAACAGGCAATTCCGCCTCGTGATGCAGGCGGTAAAGTTATTATTGTTGATATGGTGGTTGGTTATGGCGCACC
GAATCGTAAAGTTCTGGAAACCCAGGTTATGTTCGATATCTTTATCATGATGGTGAATGGTGCCGAACGTGATGAACAAGAATGGAAGAAAATCT
TTACCAAGGCAGGCTTCAAAGACTATAAAATCCTGCCGGTTCTGGGTGTGCATAGCATTATTGAAGTTTATCCGTAA

Figure S6. Codon-optimized gene sequences of Poaceae OMTs synthesized for expression in *E. coli*.
